# International practices and variability in right heart echocardiography: results from the RVNet(Work) international survey

**DOI:** 10.1186/s44156-026-00121-7

**Published:** 2026-06-08

**Authors:** Harry Magunia, Daniel X. Augustine, Attila Kovács, Alina Nicoara, Marius Keller, Dick Thijssen, Rebecca T. Hahn, Denisa Muraru, Lawrence Rudski, Karima Addetia, Monica Mukherjee, Arie van Dijk, Andre Denault, Elena Surkova, Francois Haddad, David Oxborough

**Affiliations:** 1 Department of Anesthesiology and Intensive Care Medicine, University Hospital Tübingen, Eberhard Karls University, Hoppe Seyler Str. 3, 72076 Tübingen, Germany; 2 Royal United Hospitals Bath & University of Bath, Bath, UK; 3 Department of Experimental Cardiology and Surgical Techniques, Heart and Vascular Center, Semmelweis University, Budapest, Hungary; 4 Institute for Clinical Data Management, Semmelweis University, Budapest, Hungary; 5 Department of Anesthesiology, Duke University, Durham, NC USA; 6 Department of Anesthesia and Intensive Care Medicine, Nagold Medical Center, Academic Teaching Hospital of Eberhard Karls University Tübingen, Nagold, Germany; 7 Research Institute of Sports and Exercise Sciences, Liverpool John Moores University, Liverpool, UK; 8 Department of Medical BioSciences, Radboud University Medical Center, Nijmegen, The Netherlands; 9 Columbia University Irving Medical Center/NewYork-Presbyterian Hospital, New York, NY USA; 10 Department of Medicine and Surgery, University of Milano-Bicocca, Milan, Italy; 11 Department of Cardiology, Istituto Auxologico Italiano, IRCCS, Milan, Italy; 12 Azrieli Heart Center, Department of Medicine, Jewish General Hospital, McGill University, Montreal, Canada; 13 Section of Cardiology, Department of Medicine, Heart and Vascular Center, University of Chicago, Chicago, IL USA; 14 Johns Hopkins University Division of Cardiology, Baltimore, MD USA; 15 Academic Center for Congenital Heart Disease, Radboud University Medical Center and Radboudumc Expert Center for Pulmonary Hypertension, Nijmegen, The Netherlands; 16 Department of Anaesthesiology, Montréal Heart Institute, Université de Montréal, Montréal, Canada; 17 Translational Science and Development, Cardiovascular, Renal and Metabolism, BioPharmaceuticals R&D, AstraZeneca, Cambridge, UK; 18 Royal Brompton and Harefield Hospitals, Guy’s and St. Thomas’ NHS Foundation Trust, London, UK; 19 National Heart and Lung Institute, Imperial College, London, UK; 20 Department of Medicine, Division of Cardiovascular Medicine, and Cardiovascular Institute, Stanford University, Stanford, CA USA; 21 Liverpool Centre for Cardiovascular Science at Liverpool John Moores University, Liverpool, UK

**Keywords:** Right heart, Right ventricle, Transthoracic echocardiography, Transesophageal echocardiography, Standardization, Survey

## Abstract

**Background:**

The assessment of the right heart (RH) plays a central role in the diagnosis and management of cardiovascular and pulmonary diseases. Although current guidelines have improved standardization, there is anecdotal evidence suggesting that there is significant variability in clinical practice regarding acquisition and reporting persist.

**Objectives:**

This international survey evaluated the extent of variability in echocardiographic assessment of the RH. A simple method for the standardization of RH measurements is proposed.

**Methods:**

The international anonymous survey consisted of 68 questions with a primary focus on routine methods and measurements of RH echocardiography by transthoracic (TTE) and transesophageal (TEE) modalities. The questions were developed using a standardization framework, focused on six key areas: views and settings, phase, orientation, interface, timing and selection, as well as scaling and indexing (V-POINTS).

**Results:**

The survey was available from November 2024 to February 2025. A total of 588 international respondents from various disciplines and professions responded. The majority of respondents had certification in echocardiography (74%), 34% reported 10 to 20 years of experience, 22% reported more than 20 years of experience. The 4-chamber (80%) and right ventricular (RV) focused (67%) views were most commonly used in TTE. The functional parameters included tricuspid annular plane systolic excursion (TAPSE) (80%), visual assessment (66%), RV S’ (50%), and fractional area change (FAC) (42%). RV strain and 3D metrics were less frequently used. Using TEE the mid-esophageal 4-chamber view was most commonly used (80%), RV function was mostly assessed visually, and quantification playing a secondary role (TAPSE 44% and RVFAC 34%). There was considerable variability in the definition of cardiac phases across both modalities. RV dimensions and areas were measured at the compacted region by 58% of the respondents. Scaling and indexing were not routinely used in practice.

**Conclusions:**

The survey identified significant variability in the practice of RH echocardiography. We propose a simple system (**V-POINTS) which may improve standardization of image acquisition and corresponding measurements.**

**Supplementary Information:**

The online version contains supplementary material available at 10.1186/s44156-026-00121-7.

## Introduction

The assessment of the right heart (RH) is essential for diagnosis, management, and prognostication in both chronic and acute cardiac and pulmonary diseases [1]. Echocardiography remains the frontline imaging modality for assessment of the RH in diverse clinical settings, including emergency care, perioperative monitoring, by transthoracic (TTE) and transesophageal (TEE) examinations [2]. Despite increasing recognition of the clinical importance of the RH, its evaluation remains challenging due to its complex geometry and heavily trabeculated architecture of the right ventricle (RV), as well as the limitations in acoustic windows.

Guidelines from the American Society of Echocardiography (ASE), the British Society of Echocardiography (BSE) and the European Association of Cardiovascular Imaging (EACVI) provide recommendations to improve standardization [3–5]. These societies emphasize the importance of using a dedicated RV-focused view in TTE and advocate a comprehensive assessment where appropriate, that includes tricuspid annular plane systolic excursion (TAPSE), RV fractional area change (RVFAC), strain imaging, diastolic parameters, and hemodynamic indices. In parallel, the European Society of Cardiology (ESC), BSE and ASE have proposed diagnostic algorithms for pulmonary hypertension (PH), alongside guidance for grading RV enlargement and dysfunction [3, 6, 7].

Despite published guidelines, anecdotal evidence suggests incomplete adoption of standardized RH assessment in clinical practice. Barriers may include time constraints, limited training, specific settings (e.g. pediatric or intraoperative assessment) and insufficient awareness of the prognostic significance of the RH. In addition, within the intraoperative setting, the focus of the TEE may be for evaluation of valvular lesion rather than RV quantitative analysis. Moreover, current societal guidelines do not explicitly address all aspects of standardization, such as phase selection (e.g. frame selection in systole vs. diastole and not considering ventricular dimensions, valve closure and ventricular dyssynchrony), interface selection (compacted and non-compacted regions), and optimal measurement scaling and indexing. Measurement variability is particularly evident in transverse dimensions where the position of the transverse diameter may vary significantly and there is disparate guidance from societies on the specific location of linear measurements [3]. Overall, the extent of variability in RH assessment across disciplines, professions, and geographic regions remains unknown.

**The RVNet(Work)** is a multidisciplinary collaboration of RH imaging experts committed to advancing standards, education, and research in RH assessment [8]. A central aim of the initiative is to identify gaps in clinical practice that can inform the development and integration of practical solutions [2]. These solutions should be grounded in an understanding of real-world practices and barriers, thereby enabling the creation of a roadmap for RH imaging standardization. Accordingly, the primary objective of this international survey was to evaluate routine clinical practice across healthcare disciplines in the context of RH TTE and TEE, focusing specifically on image acquisition, analysis, and interpretation. Insights from this survey are intended to support implementation of existing guidelines and to help define priorities for future research and quality improvement efforts.

## Methods

**Fig. 1:**
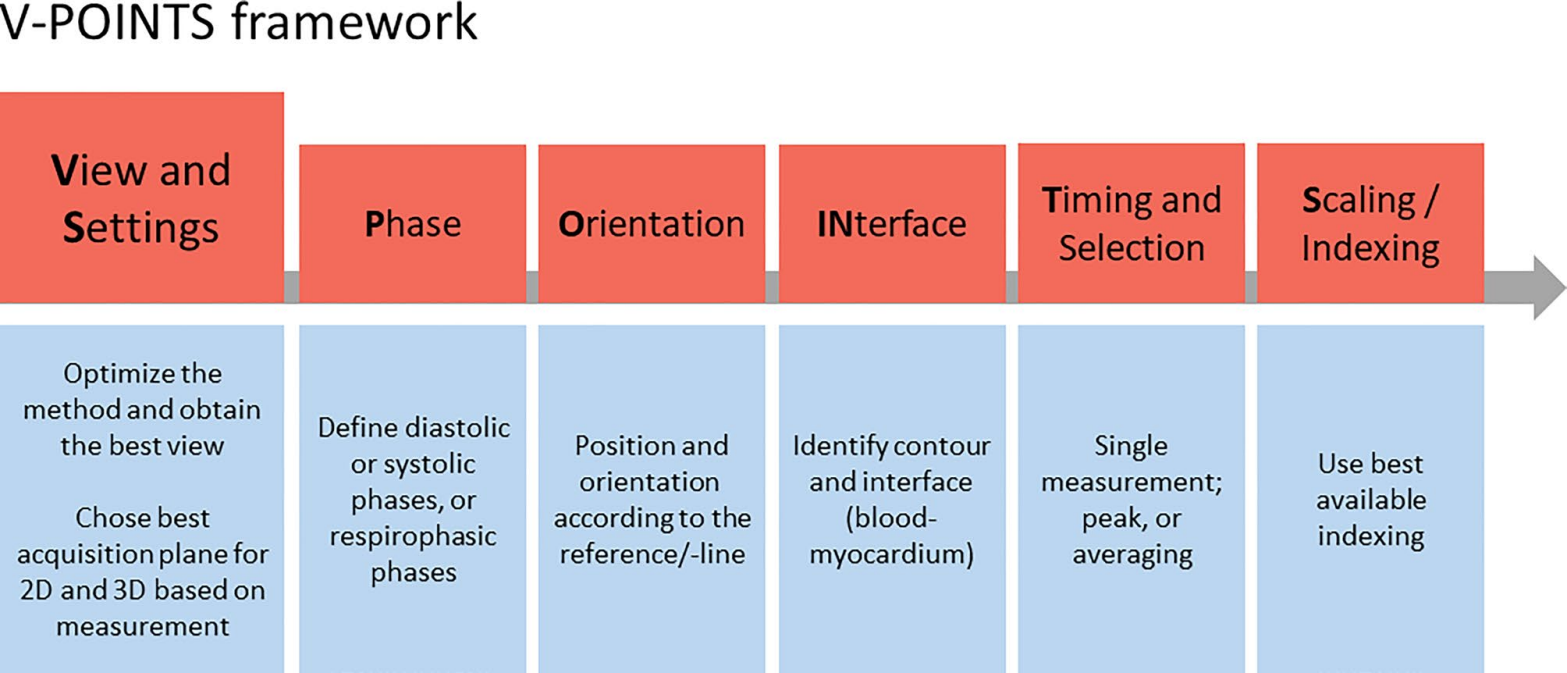
Shows the key elements of the V-Points standardization framework

The survey was created by members of the RVNet(Work) and conducted using REDCap (Research Electronic Data Capture) developed and maintained by Vanderbilt University (Nashville, USA) [8, 9]. As a first step, relevant international guidelines with content on RH imaging were screened and key questions identified. These were discussed within the RVNet(Work) and a cursory review of the variability in own practice and laboratories was carried out. While the societal guidelines [3–5, 10] should improve practice, it was apparent that variability in clinical practice may stem from elements that are either inconsistently addressed or omitted across existing guideline recommendations. To support the development of the questionnaire, we applied a bespoke principle, by first establishing a structured framework and then assessing variability across guideline recommendations [4, 10–14]. This analysis, presented in Table 1, includes the recently released ASE 2025 guidelines document [3], which was not yet available at the time the survey was initially designed. Through this process, we identified key domains contributing to variation in RH assessment. These domains formed the basis of the proposed standardization framework, summarized by the acronym **V-POINTS**: **Views and settings**, **Phase**, **Orientation**, **INterface**, **Timing and selection**, and **Scaling/Indexing (**Fig. 1**)**. This conceptual model informed both the design of the survey and the interpretation of observed practice variability.

**Table 1:** Comparison of guidelines regarding right heart measurements

2-dimensional and 3-dimensionale measurements	
**Views**	*RV linear dimensions* RV-focused view. For RV-LV basal ratio, the BSE 2020 [4] recommends the standard apical 4-chamber view. *RVOT* PLAX and PSAX. BSE 2020 and ASE 2025 [3] include PSAX bifurcation view (RVOT2). *RV areas* RV-focused view recommended. *RV volumes* WASE 2023/2024[13, 11] and ASE 2025: RV focused views ASE 2025 adds subcostal view as option *RV wall thickness* Subcostal view recommended. *RA area* Four-chamber view recommended. WASE emphasizes RA-focused views. *Inferior caval vein* Subcostal view.
**Phase**	End-diastole or end-systole – depending on measurement. WASE defines phase as follows: End-diastole: just before TV closure or onset of R wave End-systole: just before TV opening or the smallest cavity size.
**Orientation / Position**	*RV linear dimensions* Parallel measurements to the annulus. ASE 2010 [10] and BSE 2020: basal level in the basal third and mid-cavitary at the level of the LV papillary muscles. WASE [12]: basal level as the maximal third, and mid-level often occurring lower than the mid-cavitary point. ASE 2025 [3]: basal level just below the TV and the mid-level as halfway between the RV and the RV apex. *RVOT* BSE 2020 and ASE 2025 define RVOT1 and RVOT2 with precise landmarks. *RV wall thickness* ASE 2010 refers to the anterior to the TV leaflet tip; ASE 2025 recommends RV inflow region (1/3 between TV and papillary muscle). *Inferior caval vein* ASE 2025 defines 0.5–3.0 cm from RA ostium; BSE uses 1–2 cm
**Interface / Contour**	*RV linear dimensions*,* RV areas* ASE 2010: endocardial border beneath trabeculations. ASE 2025: trabeculations, moderator band, and papillary muscles should be included in the measurement. *RV wall thickness* ASE 2010 refers to the anterior to the TV leaflet tip. ASE 2025 recommends RV inflow region. *RA area* BSE 2020 and WASE recommend excluding appendages, TV tenting, IVC/SVC. *Inferior caval vein* No clear guideline
**Scaling**	*RV linear measurements* Absolute measurements, WASE presents values indexed by BSA and height *RVOT*,* RV wall thickness.* Absolute measurements. *RV areas* ASE 2010 and ASE 2025 use BSA indexing, WASE adds indexing by height. *RV volumes* WASE 2023/2024 and ASE 2025 use BSA indexing
**Doppler measurements**	
**TR velocity**	Peak velocity. ASE 2010 and BSE: recommend modal frequency with 100 mm/s sweep speed. ASE 2025 emphasizes the modal frequency, cautions use in arrhythmias
**Tricuspid Inflow**	BSE recommends averaging; WASE [14] specifies quiet breathing at end-expiration.
**TDI RV s’**	All mention angle < 20°. Modal frequency not always clearly defined. ASE 2025: 4 mm sample volume
**TAPSE**	BSE 2020 describes alignment and leading-edge technique; ASE 2025 omits specific edge definition.

2D: two-dimensional; 3D: three-dimensional; ASE: American Society of Echocardiography; BSA: body surface area; BSE: British Society of Echocardiography; IVC: inferior vena cava; LV: left ventricle; PLAX: parasternal long axis view; PSAX: parasternal short axis view; RA: right atrium; RV: right ventricle; RVOT: right ventricular outflow tract; SVC: superior vena cava; TV: tricuspid valve; WASE: World Alliance Societies of Echocardiography

The survey consisted of 68 questions, divided into four main sections: (1) basic respondent information (2), area of practice and level of expertise (3), routine methods including image acquisition, analysis, and reporting/interpretation, and (4) approaches to specific pathologies [Supplemental document]. Question types included simple binary responses (yes/no) and multiple-choice formats, minimizing the use of open text entries. It was assumed that respondents routinely optimized image quality by adjusting ultrasound machine settings; therefore, this aspect was not directly addressed in the survey. The survey was open from 18 November 2024 to 18 February 2025 and was distributed internationally via social media platforms (LinkedIn and X formerly Twitter), professional organizations, and direct email outreach by group members. Although participation was entirely anonymous, respondents were asked to provide consent for data processing. The survey complied with ethical requirements of participating countries and was registered as a National Audit in the United Kingdom and received approval from the Clinical Audit Committee of Liverpool Heart and Chest Hospital (Audit ID: CA100146).

Survey responses were exported from REDCap and analyzed using IBM SPSS Statistics (Version 30, Armonk, NY, USA) and Microsoft Excel (Version 365, Microsoft Corp., Redmond, WA, USA). Visualizations were generated using Microsoft PowerPoint (Version 365) and GraphPad Prism (Version 10.1.1, GraphPad Software Inc., Boston, MA, USA).

## Results

### Respondents

**Fig. 2:**
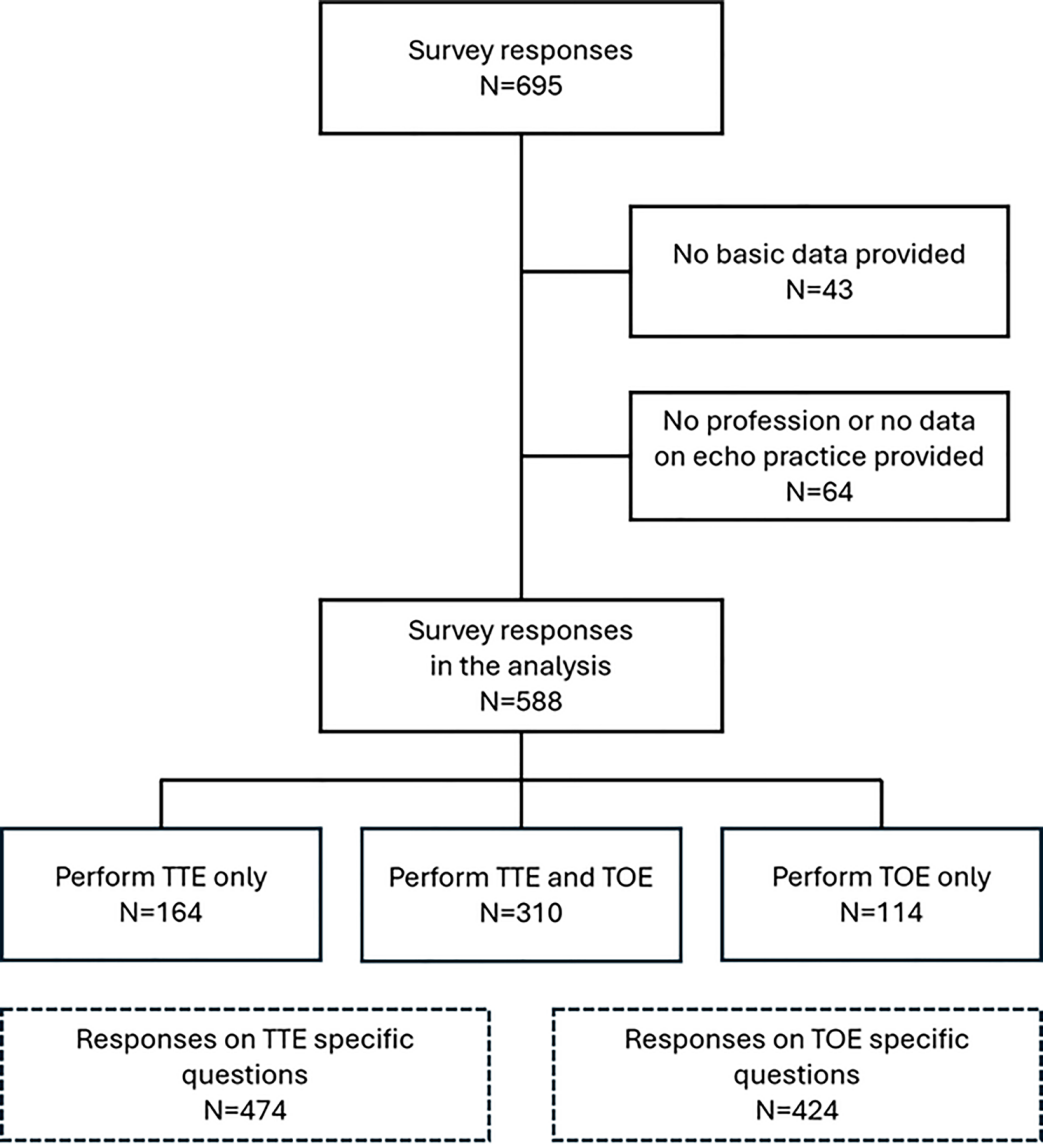
Survey response chart. TTE: transthoracic echocardiography; TOE transoesophageal echocardiography

Of the 695 respondents who accessed the survey, a total of 588 analyzable data records were collected during the study period (Fig. 2). All questions were answered by 399 (68%) of respondents. The respondents worked in 51 different countries (Supplemental Fig. 1) from a range of professional backgrounds within healthcare or academic institutes (Table 2). In 49% of cases the echo labs or hospitals were accredited for echocardiography. 74% of respondents stated that they were certified in echocardiography, 34% had been performing echocardiography between 10 and 20 years and 22% longer than 20 years. The frequency of use of TTE and TEE echocardiography varied between the different specialties, for example 98% of general non-invasive cardiologists but only 49% of respondents from the perioperative care setting perform TTE (Supplemental Table 1). There was a total of 474 datasets in response to the TTE-specific questions and 424 datasets in response to the TEE-specific questions.

**Table 2:** Basic statistics on respondents *N* = 588

Variable	*N* (%)
**Gender**	
Female	220 (37.2)
Male	360 (61.2)
Do not want to tell	8 (1.4)
**Profession**	
Physician	477 (81.1)
Sonographer	103 (17.5)
Researcher	8 (1.4)
**Area of practice**	
General non-invasive cardiology	251 (42.7)
Peri-operative care and anaesthesia	201 (34.2)
Intensive care setting	41 (7.0)
Invasive echocardiography (procedures)	39 (6.6)
Congenital heart disease	33 (5.6)
Pulmonary artery hypertension specialist	18 (3.1)
Inherited cardiac conditions / Sports cardiology	5 (0.9)
**Years of echo expertise**	
greater than 20 years	127 (21.6)
10–20 years	201 (34.2)
5–10 years	148 (25.2)
less than 5 years	112 (19.0)
**Personal accreditation/certification**	
Echo board certification	238 (40.5)
Accreditation by Echo society	197 (33.5)
Neither nor	153 (26.0)
**Setting**	
Academic hospital	369 (62.8)
Non-academic hospital	143 (24.3)
Outpatient	71 (12.1)
Clinical research laboratory	5 (0.9)
**Laboratory / Hospital accreditation**	
Yes	289 (49.1)
No	189 (32.1)
Unknown	64 (10.9)
Not applicable	446 (7.8)

### Transthoracic echocardiography: modality, views, and measurement practices

**Fig. 3:**
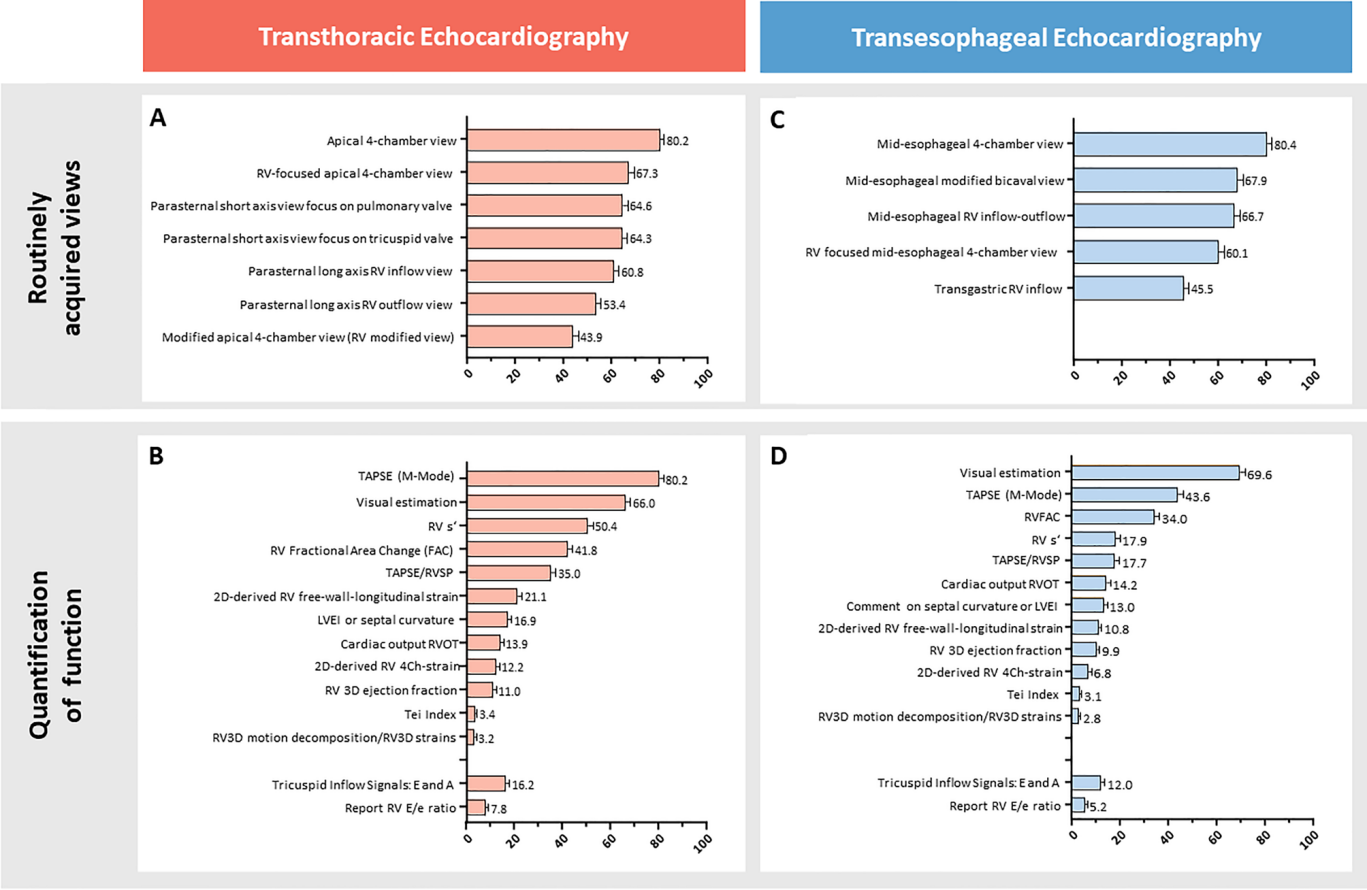
**(A)** Results showing routinely acquired views by TTE. **(B)** Results showing techniques used to quantify RV function by TTE. **(C)** Results showing routinely acquired views by TEE. **(D)** Results showing techniques used to quantify RV function by TTE

Two-dimensional TTE (2D TTE) remained the most used modality for RH imaging with the apical 4-chamber view being most commonly acquired (80%), followed by the RV-focused view (67%) (Fig. 3A). Parasternal views were used less frequently (65%). Although 65% of respondents reported having access to a 3D echocardiography probe, only 4% indicated the use of a lab-specific protocol for 3D RV imaging. Additionally, only 10% of respondents reported using electronic beam steering technologies (e.g., Philips iRotate) to optimize RV-focused views, highlighting the limited adoption of advanced image optimization tools in routine practice.

### Measurements obtained

#### RV dimensions

Linear and area measurements were undertaken and reported in 38% and 34% of examinations, respectively. 3D volumetry was rarely performed (12%) (Supplemental Table 2). RV free wall thickness was evaluated by 31% of respondents.

#### RV function

The most frequently used parameter to routinely assess RV systolic function was TAPSE made by M-mode (80%) (Fig. 3B). This was most often acquired in the standard apical 4-chamber view (41%), but also in modified apical views optimized for annular excursion (29%) and the RV-focused view (25%). Anatomical M-mode with angle correction was rarely used (5%). Visual assessment of RV function was relatively common (66%). 2D- derived RV free wall longitudinal strain (FWLS) and RV 3D ejection fraction (EF) are seldom performed, by 21% and 11% of the respondents, respectively. TAPSE/RVSP as an estimate for RV-PA-coupling is used more frequently (35%). Assessment of diastolic function was also infrequent: only 16% of respondents routinely measured tricuspid inflow E/A ratios and just 8% assessed the RV E/e′ ratio.

#### Venous assessment

Measurement of the inferior vena cava (IVC) and collapse index was widely routinely practiced (75%), while hepatic vein flow spectral Doppler was used by 46% of respondents (Supplemental Table 3). Advanced techniques such as venous excess ultrasound (VExUS) scoring were rarely adopted overall (8%) but were more frequently used in perioperative (15%) and intensive care settings (24%).

#### Right atrium and tricuspid valve

RA area was assessed by 51% of respondents and RA volume by 28%, with RA area evaluation more prevalent among general cardiologists (58%) compared to anesthesiologists (24%). RA strain was rarely used (5%). When available, tricuspid regurgitation (TR) severity was graded by 84% of respondents, and 53% measured the vena contracta.

#### Pulmonary hemodynamics

RV systolic pressure (RVSP) was estimated by 78% of respondents only. Pulmonary artery (PA) flow profiles, including acceleration time, were evaluated by 58%, whereas Doppler assessment of pulmonary valve regurgitation to estimate mean and diastolic PA pressures was less commonly performed (55% in congenital cardiology, 32% in cardiology and 18% in anesthesiology).

### Transesophageal echocardiography: views, measurements, and practice patterns

There was evidence of abbreviated protocols for the acquisition of standard TEE RV views. The most frequently obtained view was the mid-esophageal 4-chamber (80%), followed by the mid-esophageal modified bicaval view (68%) and the mid-esophageal RV inflow–outflow view (67%) (Fig. 3C). A protocol for 3D acquisitions was available in only 11% of responding labs.

### Measurements obtained

#### RV dimensions

A notable proportion of respondents (34%) reported that they do not routinely acquire or measure RV dimensions, areas, or volumes (Supplemental Table 4). Amongst those who do, RV end-diastolic and end-systolic areas (26%) and RV wall thickness (26%) were the most commonly assessed parameters. RV volumes using 3D echocardiography were rarely measured (11%).

#### RV function

The most frequently used method for evaluating RV systolic function was visual estimation, reported by 70% of respondents (Fig. 3D). TAPSE measurement using M-mode was less frequently performed (44%). As with TTE, assessment of RV diastolic function plays only a minor role in TEE: only 12% reported using tricuspid inflow E and A velocities, and just 5% reported measuring the RV E/e′ ratio.

#### Venous assessment

Assessment of IVC size including collapse index and hepatic vein Doppler flows were more commonly performed among respondents who routinely use TEE (Supplemental Table 5). In contrast, advanced techniques such as VExUS scoring or interlobular vein Doppler were almost never used. The right atrium (RA) was most often assessed by linear dimensions, while functional evaluation using RA strain was performed rarely.

#### Tricuspid valve and pulmonary pressures

Grading of TR when present, was performed by 82% of respondents. Measurement of the tricuspid annular dimensions was more commonly reported in the perioperative care setting (76%). Use of vena contracta for TR grading was consistent across specialties (77% in invasive cardiology, 64% in anesthesiology and 50% in general cardiology). Estimation of RVSP using TR was reported by 63% of respondents, whereas estimation of mean or diastolic pulmonary artery pressures was less frequently performed.

### Common aspects of standardization with focus on phase, orientation, interface, timing (selection and averaging) and scaling

Across modalities, 68% of respondents routinely documented the quality of echocardiographic images, while 59% reported on Doppler signal quality. When assessing RV shape and geometry, 72% of respondents relied on visual estimation. Only 6% reported using quantitative measurements and 21% did not report RV shape at all. Regional wall motion abnormalities, including McConnell’s sign, were recognized and reported by 71% of respondents. Use of automated non-strain segmentation software for RV analysis was limited to only 11%, reflecting low adoption of advanced computational tools (Supplemental Tables 7, 8, 9 and 10).

#### Phase

There was substantial variability in the reported definition of cardiac phases (Table 3). End-diastole was most frequently defined as the frame with the largest RV cavity (33%), followed by closure of the tricuspid valve (TV, 24%) while ECG-based definitions (R waves or T waves) were less common. Systole was most often defined as the frame with the smallest RV cavity (51%), while 28% used the frame before TV opening. Few respondents considered the role of septal dyssynchrony in assessing the RV phase.

**Table 3:** Variability in echocardiographic practice regarding definition of cardiac Phases, Orientation of measurements, INterface definition and Scaling/indexing (POINTS)

Variable	%
**Definition of end-diastole**	
Largest right ventricle	33.3%
At the tricuspid valve closure	23.6%
One frame before the tricuspid valve closure	22.3%
At the beginning of the QRS	11.5%
At the peak of the QRS	5.5%
Highest lateral tricuspid annular plane	2.4%
Other combination	1.3%
**Definition of end-systole**	
Smallest right ventricle	50.6%
One frame before the tricuspid valve opening	28.3%
At the tricuspid valve opening	12.8%
Lowest lateral tricuspid annular plane	1.5%
None of the above	6.8%
**Orientation: centerline for transverse measurements (**Fig. 4A**)**	
Linear centerline (from mid annulus to RV apex) as usually done	40.7%
A non-linear centerline that follows the curvature of the right ventricle (similar to what is done for the aorta or coronary arteries)	33.4%
Parallel measures to the tricuspid annulus independent of the centerline	23.2%
Other	2.7%
**Orientation: RV apex definition (**Fig. 4B**)**	
Apex positioned at the septal junction	54.3%
Apex defined as the maximal distance from the right ventricular centroid to the RV contour	31.1%
Response “I don’t know”	14.6%
**Interface definition: blood-myocardium border (**Fig. 4C**)**	
At the non-compacted region	39.4%
At the compacted region	58%
Other	2.7%
**Interface definition: use of modal frequency (**Fig. 4D**)**	
Blood Doppler	80.6%
Tissue Doppler	76.5%
**Scaling and indexing of diameters**	
No-indexing	67%
Index to BSA	30%
Other	3%
**Scaling and indexing of areas**	
No-indexing	59%
Index to BSA	40%
Other	2%
**Scaling and indexing of volumes**	
No-indexing	51%
Index to BSA	47%
Other	2%

BSA: body-surface area; RV: right ventricle. %: Percentage of positive responses

#### Orientation

**Fig. 4:**
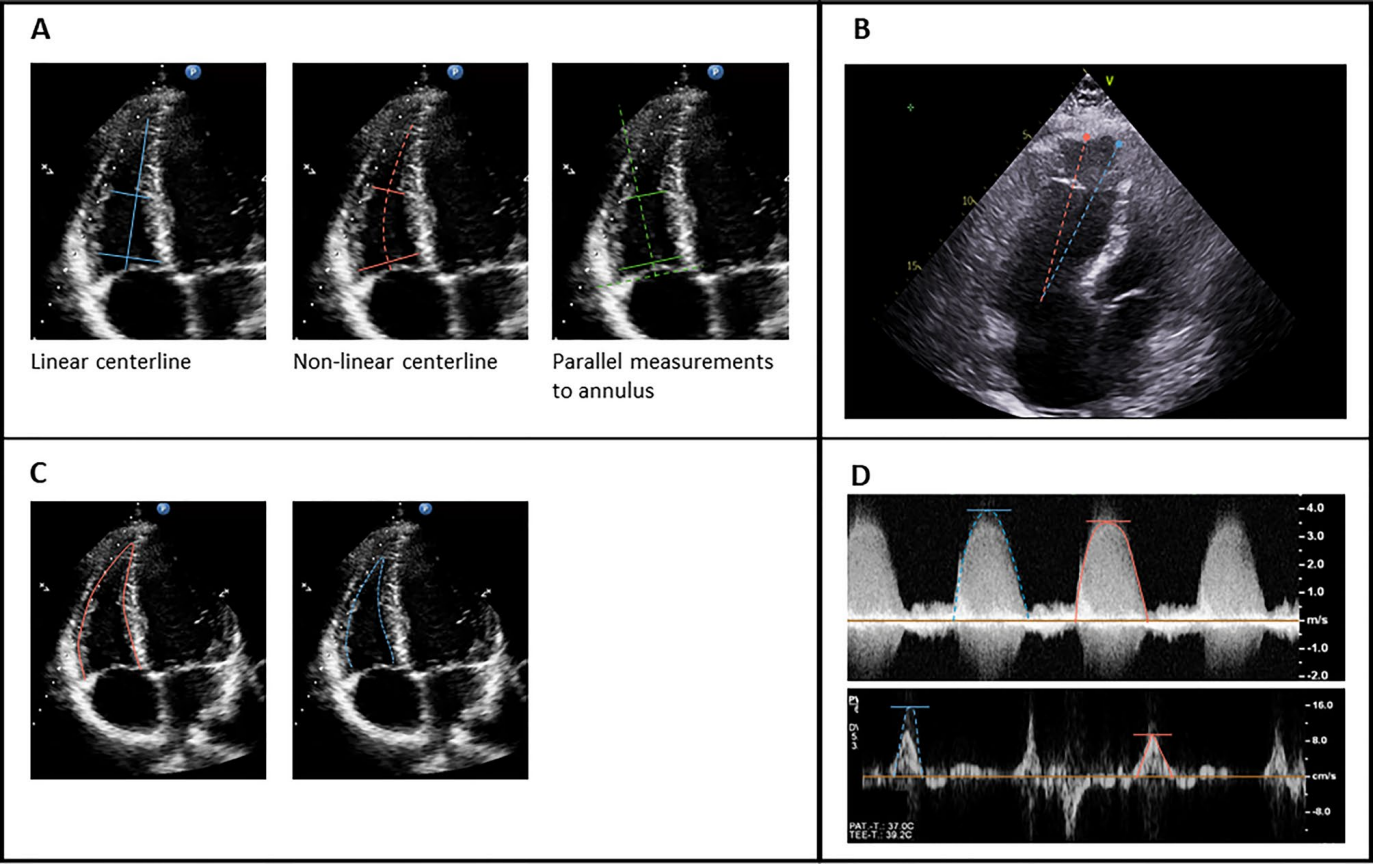
Echocardiographic images related to standardization of measurements - all of them were used in the survey. **(A)** Definition of centerline for transverse measurements. Left: Linear centerline (from mid annulus to RV apex; blue); Middle: A non-linear centerline that follows the curvature of the right ventricle (similar to what is done for the aorta or coronary arteries (red): Right: Parallel measures to the tricuspid annulus independent of the centerline (green). **(B)** Definition of RV apex: Blue dotted line: apex positioned at the septal junction; red dotted line: apex defined as the maximal distance from the right ventricular centroid to the RV contour. **(C)** Definition of endocardial/blood-myocardium border: left - at the compacted region (red); right – at the non-compacted region. **(D)** Use of modal frequency for Doppler measurements. Upper image: continuous-wave blood Doppler (red line: modal frequency; blue dotted line: maximal velocity). Lower image: tissue spectral Doppler (red line: modal frequency; blue dotted line: maximal velocity)

There was significant variability in the methods used for assessing diameters with 41% favoring alignment to a linear centerline approach. 33% favored alignment to a non-linear centerline that follows RV curvature and 23% favored parallel measurements relative to the TV annulus (Table 3; Fig. 4A). The RV apex was most frequently defined at the septal junction (54%), 31% defined it as the point furthest from the RV centroid and 15% stated they were uncertain or unaware of the definition (Fig. 4B).

#### Interface

The interface between myocardium and blood pool was variably defined: 58% of respondents measured at the compacted myocardial border while 39% included part of the non-compacted region (Fig. 4C). 3% used alternative definitions (e.g., 1–2 mm into the non-compacted zone) (Fig. 4D). In terms of Doppler analysis, the modal frequency (selecting the best-defined Doppler envelope) was applied by 81% of respondents for blood flow Doppler measurements and 77% for tissue Doppler evaluations.

#### Scaling and indexing

Although it is well established that cardiac chamber size is directly associated with body size, the integration of scaling methods are not used in routine practice. (Table 3). Linear dimensions are most often reported as absolute values. Regarding indexing practices for RV parameters: linear diameters were not indexed by 67% of respondents, area measurements were not indexed in 59% and volume measurements were not indexed in 51%. Where indexing was performed, body surface area (BSA) was the most common reference standard. Other scalars of body size (including height) or the adoption of other allometric coefficients were rarely applied.

### Grading and pathology

#### Grading systems for RV function

56% of respondents used a standardized system or threshold to assess global RV function. Grading was more consistently applied for specific metrics, TAPSE (79%), RV FAC (60%), FWLS (39%), 3D EF (27%). Notably, 44% of respondents stated that they use the same grading system regardless of patient context (e.g., athletes, surgical patients). 31% reported having institutional thresholds to define meaningful changes in RV function or RVSP (Supplemental Table 11).

## Discussion

Despite ongoing efforts by professional societies to improve the standardization of echocardiographic assessment of the RH, our survey highlights persistent and significant variability in clinical practice. This variability spans several key domains, including the definition of cardiac phases (systole and diastole), anatomical orientation, myocardium–blood pool interface, signal selection for Doppler and tissue imaging, and indexing methods. While these elements are addressed in existing guidelines, the level of detail and consistency across documents remains incomplete, which may contribute to challenges in implementation. In addition to the fields of diagnostic and interventional cardiology, we also demonstrate how RH echocardiography is applied in the perioperative and intensive care settings.

### Transthoracic echocardiography

2D-TTE is the first-line echocardiographic investigation for the assessment of the RH due to its ability to comprehensively image both the RV and the RA. There are however inherent limitations associated with imaging the complex RV structure using a transthoracic approach and hence there is a need for standardization of both acquisition and analysis. Several societal guidelines have been published to try to address this. The results of our survey highlight, that in part, these guidelines are not being fully implemented. A comprehensive assessment of RV structure and function requires multiple views to ensure full representation of the RV and RA and to capture overt or subtle changes in function [15]. Societies and professional bodies clearly highlight the need for a minimum dataset of images [16, 17]. This is particularly important in specific disease states where diagnosis and subsequent monitoring is key for optimal patient management [18–20]. There is a significant risk of misdiagnosis and / or lack of timely therapy or intervention if imaging is not comprehensive [21, 22].

The clinical demand for TTE continues to rise, with growing pressures on the echocardiographic workforce. At the same time, new advanced echocardiographic parameters and techniques (GLS, 3D volumetric analysis, atrial strain etc.) are being introduced, adding complexity. These competing demands often lead to abbreviated examinations to improve throughput creating tension between efficacy and quality. This is likely a major contributor to reduced implementation of guidelines recommended practices and should be a priority for professional bodies and societies. Evidence suggests that a comprehensive TTE may require 45–60 min and therefore it is important that echocardiography departments plan capacity accordingly [23]. It should also be noted that in time-critical emergency situations, prolonging examinations can be impractical and unsafe. In these settings, it is important to prioritize imaging that will guide immediate therapy and intervention, with limited acquisitions and measurements under established caveats. Targeted societal guidance, outlining a prioritized protocol in urgent settings is beneficial but should not replace comprehensive TTE assessment when feasible [24].

When fewer images are acquired, the downstream impact is significant as fewer measurements can be provided. This is reflected in our survey where fewer than 50% of the respondents reported consistently linear dimensions, areas, and functional parameters. These rates are notably lower than those reported in a 2022 survey, which was conducted exclusively in certified centers including a high rate of tertiary-care centers [25]. Interestingly, over 66% of respondents favored a qualitative visual assessment for chamber size and function, lower than the rates previously reported [26]. This difference may, in part, be due to the different recruitment structure of the 2019 survey. The survey was conducted among users of an online learning platform, 64% of whom were beginners or moderately advanced learners. In contrast, the dataset presented here consists predominantly of responses from experienced examiners [26]. While qualitative approaches are faster, these are limited by poor validity and reproducibility, potentially affecting patient care [27–29]. Previous studies have highlighted the considerable variability in subjective assessment, largely dependent on operator experience [30]. Furthermore, limited image acquisition constrains qualitative or quantitative assessment. It is therefore not surprising that TAPSE has emerged as the most frequently reported functional parameter due to its rapid acquisition and minimal reliance on acoustic windows. Also, multiparametric approaches often lead to uncertainty when results based on various parameters are not concordant [31]. In these situations, clinicians tend to rely on more traditional parameters. Further research studies aimed at highlighting the reproducibility, methodology and clinical relevance of quantitative changes are essential in providing the echocardiographer with confidence and assurance that quantitative parameters are valuable and impactful in decision-making [32].

Newer techniques such as 3D and strain imaging have been shown to have important diagnostic and prognostic value [33–35]. Our international survey underlines the findings by other Europe and UK-focused surveys [36, 37], that, with exception of very specific clinical settings i.e. for interventional assessment of the tricuspid valve [38], there is a translational lag of integration into clinical practice (11% of respondents using 3D RV volumes and 21% for RVFWS). With the development of automated techniques and faster post-processing capabilities these modalities are becoming easier to use with enhanced validity and reproducibility. There is a drive from manufacturers to increase implementation, and we anticipate that with continual education and robust research data demonstrating diagnostic and prognostic value, these techniques will become available for routine investigations and will be integrated routinely.

### Transesophageal echocardiography

As part of a comprehensive TEE examination, assessment of ventricular function in addition to valve pathologies is essential. The current ASE guidelines highlight the role of TEE in evaluation of RH chambers, particularly when TTE image quality is limited [39]. Unfortunately, there is still no guideline that proposes a strategy for quantifying RV function using TEE in sedated or ventilated patients. Anatomically, TEE offers the advantage of visualizing an enlarged RV, as it can be fully captured in the far-field of the imaging sector. Despite this advantage, our survey shows that, like the transthoracic modality, all recommended sectional planes are rarely captured. This finding contrasts with existing guidelines, which specifically emphasize the importance of incorporating multiple views [39, 40].

In intraoperative and intensive care settings, TEE is often the only option for evaluating cardiac function. In these settings, time-critical decisions are frequently required, therefore adequate RV evaluation is furthermore paramount. A comprehensive TEE examination under sedation usually requires around 30 min and should include all recommended imaging planes for assessment of the RV. These images can then be quantified post-processing. In the operating room, where a TEE may extend over several hours of surgery, it is feasible to perform a comprehensive evaluation of RV function both before and after the surgical procedure. Because RH function plays a critical role during cardiac surgery, transient RV systolic dysfunction may be common in the early peri-operative period and hence a comprehensive examination enables better tracking of subsequent changes and supports interpretation of postoperative hemodynamic instability [41].

TEE examination appears to remain largely reliant on visual assessment. Our survey shows that linear dimensions and areas are measured infrequently. In addition to visual estimation of RV function with limited validity [27, 28], only TAPSE by M-mode and RV FAC are used to any significant extent. However, the use of TAPSE by TEE is debated. These measurements are not directly comparable with those obtained with TTE and their utility in the perioperative setting remains questionable [9–12]. Advanced techniques, such as strain analysis and 3D quantification, are used in fewer than 10% of cases, despite their established prognostic value. The time-consuming post-processing analysis required may limit their adoption, though AI-based tools have the potential to overcome this barrier in the future.

### V-POINTS standardization proposal

We demonstrate a considerable degree of variability regarding standardization of measurements. This is evident in several areas including phase definition, delineation of blood-myocardium interface, and use of modal frequency in Doppler measurements. Parameter scaling is particularly relevant for areas and volumes, and we have observed that adjustment for body-surface area (BSA) are applied infrequently. Regarding linear dimensions, most respondents preferred a linearly constructed centerline. Ultimately, our survey shows that, contrary to scientific literature [1], disease-specific scoring systems are not routinely applied in clinical practice.

In the past 15 years, echocardiographic societies, have significantly advanced clinical practice for RH evaluation by publishing guideline documents [3–5, 10]. These efforts have not only improved image acquisition protocols but also defined reference limits to use in practice. Table 1 summarizes the major recommendations across societies using the V-POINTS framework.

For 2D RV linear and area measurements, all guidelines consistently recommend performing assessment at end-diastole and end-systole using the RV-focused view. However, the exact definition of these phases is not always clearly specified. The World Alliance Societies of Echocardiography (WASE) initiative uniquely provides a more rigorous phase definition, referencing TV closure and R wave on ECG [12]. This may be especially important in conditions such as pulmonary hypertension or congenital heart disease, where RV contraction is often dyssynchronous [42, 43]. Accurate phase definition is equally critical for timing measurements such as RV inflow or tissue Doppler parameters. For instance, the BSE recommends signal averaging, while WASE advocates for measurements at end-expiration - highlighting both physiological and methodological variability [4, 14].

Perhaps one of the greatest sources of variation lies in the definition of RV basal and mid transverse diameters. In both the BSE2020 guidelines and the WASE study, the RV basal diameter is defined as the maximal dimension parallel to the annular plane (in the basal third of the RV) [4, 12]. In contrast, ASE2025 redefined this as the diameter immediately below the tricuspid valve which may introduce variability secondary to RV basal curvature [3]. Similarly, definitions of the RV mid-transverse diameter vary: BSE and ASE2010 guidelines reference the level of the LV papillary muscles, WASE uses the maximal diameter, and ASE2025 guidelines defines it as a region in the mid-RV, lacking clear anatomical landmarks. Some of this variability might be reduced by adopting a centerline-based approach, like those used in engineering, where transverse diameters are measured perpendicular to a reference axis. However, this will depend on future validation and vendor implementation.

The RV blood-myocardium interface is often challenging to clearly visualize given the highly trabeculated anatomy. For example, in 2D and 3D imaging, the boundary between blood pool and myocardium (endocardial border) is often not clearly defined, contributing to measurement variation currently not consistently specified. In addition, the trabeculated RV myocardium compounds this issue through an inability to clearly differentiate the compacted and non-compacted regions. Therefore, for 2D imaging, the focus should likely be only on excluding trabeculations and using annular landmark for basal boundaries, However, for 3D imaging, this approach may lead to an underestimation of ventricular volumes. Particularly for 3D echocardiography, there is data showing a high variance in measurements compared to cardiac MRI of the RV, which can be explained, in part, by the definition of the endocardial border [44]. Additionally, heavy trabeculations in the apex and the moderator band must be identified to correctly measure dimensions and areas [10]. In Doppler imaging, although guidelines often recommend using the modal frequency, many published illustrations and clinical reports appear to measure outside this range [45]. Choosing the best signal for reporting is critical. Several RH parameters recommend using peak values, such as in TR velocity or tissue Doppler imaging. While these can capture the maximum physiological response, they may also lead to overestimation particularly in individuals with marked respiratory change.

Scaling and indexing are essential to avoid misclassification of chamber size [3, 4, 12]. Ideally, a scaling method should result in a body-size independent parameter with no residual association between the indexed value and the scaling parameter—not only for mean values but also for the limits of the distribution (e.g., reference limits). Beyond height and weight, effective indexing can also incorporate sex, race, and age adjustments to improve equity and accuracy. Until recently, there has been a paucity of patient-level scaling data but the publication of WASE data, among others, has now provided workable values that allow for indexing [14]. Further work is required to provide the evidence supporting the correct scalar and whether a ratiometric or an allometric approach should be favored [46, 47].

#### A step forward

To address these gaps, and in collaboration with expert groups across multiple societies, we propose the adoption of a more structured and unified approach through the V‑POINTS framework (Fig. 1). This model highlights seven core domains essential to RH standardization: Views, Phase, Orientation, Interface, Timing, Scaling, and Setting. By explicitly specifying the elements that should be defined and reported, V‑POINTS offers a practical roadmap to reduce ambiguity, enhance reproducibility, and support harmonized implementation of RH measurements in both clinical and research settings.

#### Limitations

This survey represents a snapshot in time and has likely not captured evolving practices or the impact of newer guidelines such as the recently published ASE 2025 guidelines. The sample size, while adequate for exploratory insights, remains relatively modest and may not fully reflect practices in broader community or non-specialty settings. These latter setting may face greater pressures to perform abbreviated studies. Accordingly, with a higher percentage of academic centers, this may mask an even lower implementation of societal recommendations to perform quantitative analyses. Additionally, the survey relied on self-reported practices, which may be subject to recall bias or reflect idealized rather than routine behavior. The cross-section of participants may be skewed by the fact that they were recruited via various professional society mailing lists and may not provide a complete representative sample of those who perform echocardiography. Unfortunately, the data does not allow for certain specific analyses, such as whether the low number of linear measurements is due to the absence or impossibility of capturing the necessary cross-sections. The fact that sonographers perform echocardiography in some countries may have led to varying results, which could be examined in more detail in future focused surveys. Despite these limitations, this survey is the first of its kind and provides meaningful insights into current variability and key areas of uncertainty in RH imaging practice by TTE and TEE.

## Conclusion

This international survey spanning both academic and non-academic institutions, demonstrated substantial heterogeneity in RH assessment, particularly in the application of basic measurement principles. A standardized framework for image acquisition, processing and measurement such as the **V-POINTS framework used in this survey**, may support improved standardization across clinical settings and international guidelines. This approach will need to be supported by a robust knowledge-to-action program which would be best supported through national and international medical societies.

## Electronic Supplementary Material

Below is the link to the electronic supplementary material.

Supplementary Material 1

Supplementary Material 2

## Data Availability

The datasets used and/or analysed during the current study are available from the corresponding author on reasonable request.

## Abbreviations

ASE: American Society of Echocardiography
BSA: Body Surface Area
BSE: British Society of Echocardiography
EACVI: European Association of Cardiovascular Imaging
ESC: European Society of Cardiology
IVC: Inferior vena cava
PA: Pulmonary Artery
PH: Pulmonary Hypertension
RA: Right Atrium
RH: Right Heart
RV: Right Ventricle
RVFAC: Right Ventricular Fractional Area Change
RVSP: Right Ventricular Systolic Pressure
TAPSE: Tricuspid Annular Plane Systolic Excursion
TTE: Transthoracic Echocardiography
TEE: Transesophageal Echocardiography
TOF: Tetralogy of Fallot
TR: Tricuspid regurgitation
TV: Tricuspid valve
VExUS: Venous excess ultrasound
WASE: World Alliance Societies of Echocardiography
