# Supplementary material for: International practices and variability in right heart echocardiography: results from the RVNet(Work) international survey
Source: Echo Res Pract. 2026 Jun 8;13:22. doi: 10.1186/s44156-026-00121-7 (PMC13245076; doi:10.1186/s44156-026-00121-7)
Supplement: Supplementary file 1 — Supplementary Material 1 [file 44156_2026_121_MOESM1_ESM.docx]

**Supplemental Appendix**

**Table of contents**

[Study organization 2](#_Toc225765126)

[Study collaborators 3](#_Toc225765127)

[Supplemental Figure 1: Heatmap - Distribution of responses by country 4](#_Toc225765128)

[Color coding of supplemental tables 6](#_Toc225765129)

[Supplemental Table 1: Count of procedures by area of practice 7](#_Toc225765130)

[Supplemental Table 2: Dimensions and function by TTE 8](#_Toc225765131)

[Supplemental Table 3: Evaluation of Tricuspid Valve and Blood Conducting System by TTE 9](#_Toc225765132)

[Supplemental Table 4: Dimensions and function by TEE 10](#_Toc225765133)

[Supplemental Table 5: Evaluation of Tricuspid Valve and Blood Conducting System by TEE 11](#_Toc225765134)

[Supplemental Table 5: Availability of a protocol to optimize 3D acquisitions 12](#_Toc225765135)

[Supplemental Table 6: Definition of end-systolic frame in the presence of abnormal septal motion 12](#_Toc225765136)

[Supplemental Table 7: Report on image quality 12](#_Toc225765137)

[Supplemental Table 8: Reporting on RV shape /geometry 13](#_Toc225765138)

[Supplemental Table 9: Reporting on regional wall motion abnormalities of the RV (e.g. McConnel's sign, regional RV strain) 13](#_Toc225765139)

[Supplemental Table 10: 54: About automatic segmentation methods: Do you routinely use automatic segmentation methods (non strain imaging) for the right heart in the laboratory, such as autoRV (Tomtec), LVivoRV, US2.ai or others? 13](#_Toc225765140)

[Supplemental Table 11: Grading systems of RV function 14](#_Toc225765141)

# Study organization

This international survey was developed and conducted by the RVNet(Work) group.

Executive committee members of the RVNet(Work) group:

- **Daniel Augustine**; Royal United Hospitals Bath & University of Bath, Bath, UK
- **André Denault**; Department of Anaesthesiology, Montréal Heart Institute, Université de Montréal, Montréal, Canada
- **Arie van Dijk**; Academic Center for Congenital Heart Disease, Radboud University Medical Center and Radboudumc Expert Center for Pulmonary Hypertension, Nijmegen, the Netherlands
- **Francois Haddad**; Department of Medicine, Division of Cardiovascular Medicine, and Cardiovascular Institute, Stanford University, Stanford, California, USA
- **Marius Keller**; Department of Anesthesia and Intensive Care Medicine, Nagold Medical Center, Academic Teaching Hospital of Eberhard Karls University Tübingen, Nagold, Germany
- **Attila Kovács**; Department of Experimental Cardiology and Surgical Techniques, Heart and Vascular Center, Semmelweis University, Budapest, Hungary and Institute for Clinical Data Management, Semmelweis University, Budapest, Hungary
- **Harry Magunia**; Department of Anesthesiology and Intensive Care Medicine, University Hospital Tübingen, Eberhard Karls University, Tübingen, Germany
- **Alina Nicoara**; Department of Anesthesiology, Duke University, Durham, North Carolina, USA
- **David Oxborough**; Liverpool Centre for Cardiovascular Science at Liverpool John Moores University, Liverpool, UK
- **Elena Surkova**; Translational Science and Development, Cardiovascular, Renal and Metabolism, BioPharmaceuticals R&D, AstraZeneca, Cambridge, UK; Royal Brompton and Harefield Hospitals, Guy's and St. Thomas' NHS Foundation Trust, London, UK; National Heart and Lung Institute, Imperial College, London, UK
- **Dick Thijssen**; Research Institute of Sports and Exercise Sciences, Liverpool John Moores University, Liverpool, UK; Department of Medical BioSciences, Radboud University Medical Center, Nijmegen, the Netherlands

# Study collaborators

The following organizations collaborated by distributing the survey to their members.

- ASEAN Society of Echocardiography; Jose Donato Magno
- British Society of Echocardiography
- Departamento de Imagem Cardiovascular da Sociedade Brasileira de Cardiologia; Silvio Henrique Barberato
- Deutsche Gesellschaft für Ultraschall in der Medizin (DEGUM); Jan Knierim
- Deutsche Gesellschaft für Anaesthesiologie und Intenisvmedizin (DGAI); Matthias Göpfert
- Indian Academy of Echocardiography; Shantanu Sengupta
- Schweizer Gesellschaft Kardiologie AG Imaging; Felix Tanner

# Supplemental Figure 1: Heatmap - Distribution of responses by country


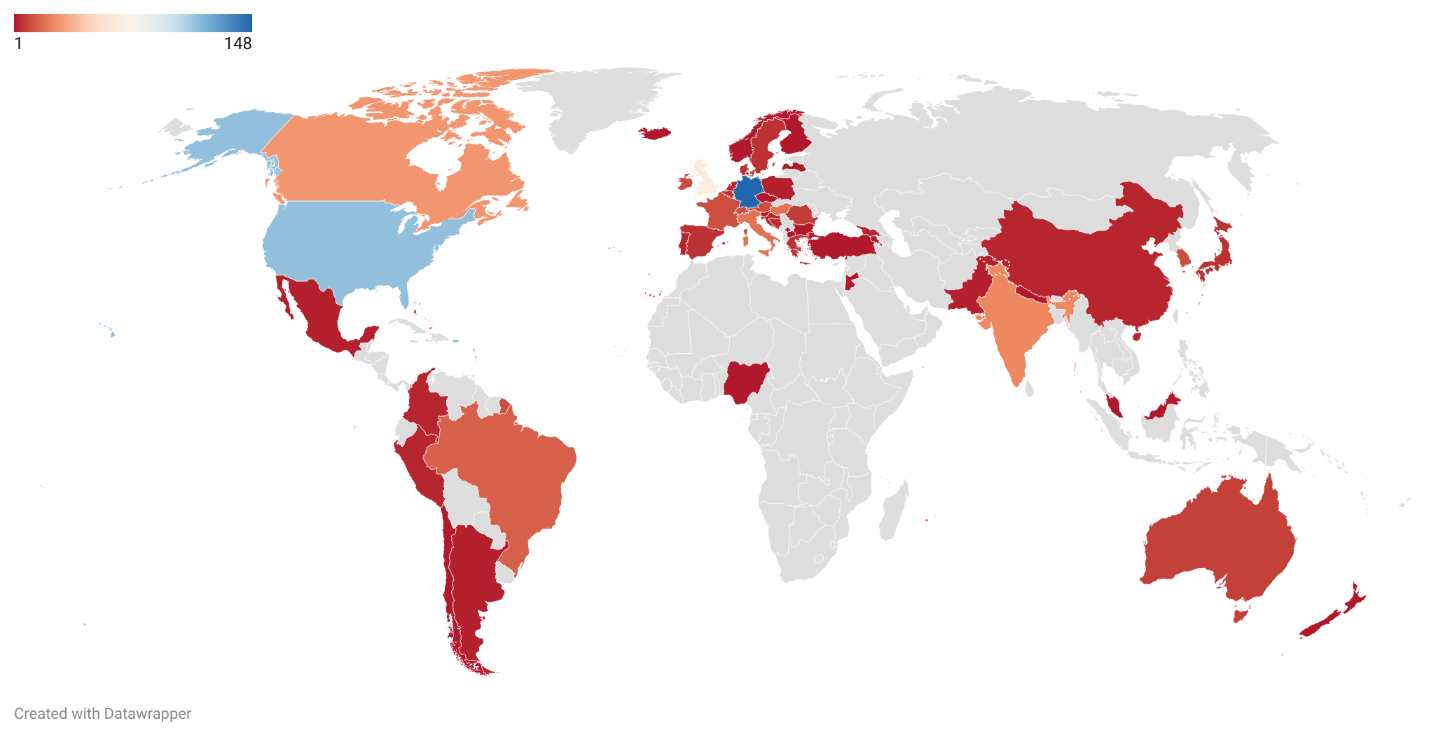


| **Country** | **Count (N)** | **Procent (%)** |
| --- | --- | --- |
| Argentina | 2 | 0,3 |
| Armenia | 1 | 0,2 |
| Australia | 8 | 1,4 |
| Austria | 11 | 1,9 |
| Belgium | 5 | 0,9 |
| Bosnia and Herzegovina | 5 | 0,9 |
| Brazil | 15 | 2,6 |
| Bulgaria | 1 | 0,2 |
| Canada | 29 | 4,9 |
| Chile | 1 | 0,2 |
| China | 3 | 0,5 |
| Colombia | 3 | 0,5 |
| Croatia | 3 | 0,5 |
| Czech Republic | 1 | 0,2 |
| Denmark | 5 | 0,9 |
| Finland | 1 | 0,2 |
| France | 11 | 1,9 |
| Georgia | 3 | 0,5 |
| Germany | 148 | 25,2 |
| Greece | 5 | 0,9 |
| Hungary | 20 | 3,4 |
| Iceland | 1 | 0,2 |
| India | 25 | 4,3 |
| Ireland | 10 | 1,7 |
| Italy | 20 | 3,4 |
| Japan | 5 | 0,9 |
| Jordan | 1 | 0,2 |
| Kosovo | 1 | 0,2 |
| Latvia | 1 | 0,2 |
| Malaysia | 1 | 0,2 |
| Mexico | 2 | 0,3 |
| Nepal | 1 | 0,2 |
| Netherlands | 3 | 0,5 |
| New Zealand | 1 | 0,2 |
| Nigeria | 1 | 0,2 |
| North Macedonia | 1 | 0,2 |
| Norway | 1 | 0,2 |
| Pakistan | 2 | 0,3 |
| Peru | 3 | 0,5 |
| Poland | 2 | 0,3 |
| Portugal | 3 | 0,5 |
| Republic of Korea | 11 | 1,9 |
| Romania | 7 | 1,2 |
| Slovenia | 1 | 0,2 |
| Spain | 5 | 0,9 |
| Sweden | 5 | 0,9 |
| Switzerland | 11 | 1,9 |
| The Bahamas | 1 | 0,2 |
| Turkey | 1 | 0,2 |
| United Kingdom | 66 | 11,2 |
| United States of America | 114 | 19,4 |
| **Total** | 588 | 100,0 |

# Color coding of supplemental tables

The tables in this supplement are color-coded using a heat map. The lowest percentage value is marked in blue, and the highest percentage value is marked in red. In between, there is a color gradient from blue to red via white (the average percentage value).

# Supplemental Table 1: Count of procedures by area of practice

|  | **Transthoracic Echocardiography** | | **Transoesophageal Echocardiography** | |
| --- | --- | --- | --- | --- |
|  | Performs Yes/Total (%) | Cases per week Median (IQR) | Performs Yes/Total (%) | Cases per week Median (IQR) |
| **General non-invasive cardiology** | 248/251 (98.8) | 47.5 (25-82.5) | 123/251 (49.0) | 4.5 (2-8.5) |
| **Peri-operative care and anaesthesia** | 98/201 (48.8) | 5 (2-8) | 196/204 (96.1) | 5 (3-10) |
| **Intensive care setting** | 41/41 (100) | 10 (5-20) | 29/41 (70.7) | 2.5 (1-5) |
| **Invasive echocardiography (procedures)** | 34/39 (87.2) | 40 (25-60) | 34/39 (87.2) | 10 (5.5-15) |
| **Congenital heart disease** | 31/33 (93.9) | 35 (30-57.5) | 22/33 (66.7) | 4.5 (1.8 – 8.5) |
| **Pulmonary artery hypertension specialist** | 17/18 (94.4) | 30 (10-100) | 12/18 (66.7) | 5 (2-13.8) |
| **Inherited cardiac conditions / Sports cardiology** | 5/5 (100) | 17.5 (n/a) | 3/5 (60.0) | 9 (n/a) |

IQR: inter-quartile range.

# Supplemental Table 2: Dimensions and function by TTE

|  | All responses N=474 | | General non-invasive cardiology N=248 | | Peri-operative care and anesthesia N=98 | | Intensive care setting N=41 | | Invasive echocardiography (procedures) N=24 | | Congenital heart disease N=31 | | Pulmonary artery hypertension specialist N=17 | | Inherited cardiac conditions / Sports cardiology N=5 | |
| --- | --- | --- | --- | --- | --- | --- | --- | --- | --- | --- | --- | --- | --- | --- | --- | --- |
|  | Count | Percent | Count | Percent | Count | Percent | Count | Percent | Count | Percent | Count | Percent | Count | Percent | Count | Percent |
| **Dimension, Areas and Volumes** | | | | | | | | | | | | | | | | |
| RVD1 only | 172 | 36.3% | 115 | 46.4% | 21 | 21.4% | 14 | 34.1% | 10 | 29.4% | 6 | 19.4% | 5 | 29.4% | 1 | 20.0% |
| RV linear dimensions inflow (RVD1, RVD2, RVD3) | 179 | 37.8% | 92 | 37.1% | 30 | 30.6% | 18 | 43.9% | 13 | 38.2% | 16 | 51.6% | 8 | 47.1% | 2 | 40.0% |
| RV linear dimensions outflow (RVOT) | 121 | 25.5% | 62 | 25.0% | 17 | 17.3% | 10 | 24.4% | 11 | 32.4% | 13 | 41.9% | 6 | 35.3% | 2 | 40.0% |
| RV end-diastolic and end-sysolic area | 159 | 33.5% | 79 | 31.9% | 25 | 25.5% | 15 | 36.6% | 14 | 41.2% | 15 | 48.4% | 9 | 52.9% | 2 | 40.0% |
| RV volumes by 3D echocardiography | 58 | 12.2% | 25 | 10.1% | 9 | 9.2% | 4 | 9.8% | 8 | 23.5% | 7 | 22.6% | 4 | 23.5% | 1 | 20.0% |
| RV wall thickness | 146 | 30.8% | 68 | 27.4% | 30 | 30.6% | 17 | 41.5% | 13 | 38.2% | 8 | 25.8% | 8 | 47.1% | 2 | 40.0% |
| None of the above | 55 | 11.6% | 19 | 7.7% | 21 | 21.4% | 6 | 14.6% | 2 | 5.9% | 7 | 22.6% | 0 | 0.0% | 0 | 0.0% |
| **Systolic and diastolic function** | | | | | | | | | | | | | | | | |
| Visual estimation of RV function | 313 | 66.0% | 158 | 63.7% | 71 | 72.4% | 34 | 82.9% | 14 | 41.2% | 27 | 87.1% | 7 | 41.2% | 2 | 40.0% |
| Tei Index | 16 | 3.4% | 9 | 3.6% | 4 | 4.1% | 0 | 0.0% | 2 | 5.9% | 0 | 0.0% | 1 | 5.9% | 0 | 0.0% |
| RV Fractional Area Change (FAC) | 198 | 41.8% | 97 | 39.1% | 35 | 35.7% | 17 | 41.5% | 18 | 52.9% | 20 | 64.5% | 10 | 58.8% | 1 | 20.0% |
| TAPSE by M-Mode | 380 | 80.2% | 213 | 85.9% | 65 | 66.3% | 33 | 80.5% | 26 | 76.5% | 26 | 83.9% | 13 | 76.5% | 4 | 80.0% |
| TAPSE/RVSP | 166 | 35.0% | 93 | 37.5% | 29 | 29.6% | 14 | 34.1% | 9 | 26.5% | 12 | 38.7% | 8 | 47.1% | 1 | 20.0% |
| 2D-derived RV 4Ch-strain | 58 | 12.2% | 26 | 10.5% | 9 | 9.2% | 3 | 7.3% | 6 | 17.6% | 7 | 22.6% | 7 | 41.2% | 0 | 0.0% |
| 2D-derived RV free-wall-longitudinal strain | 100 | 21.1% | 58 | 23.4% | 10 | 10.2% | 7 | 17.1% | 9 | 26.5% | 11 | 35.5% | 5 | 29.4% | 0 | 0.0% |
| RV 3D ejection fraction | 52 | 11.0% | 25 | 10.1% | 4 | 4.1% | 4 | 9.8% | 9 | 26.5% | 4 | 12.9% | 5 | 29.4% | 1 | 20.0% |
| RV 3D motion decomposition / RV 3D strains | 15 | 3.2% | 4 | 1.6% | 1 | 1.0% | 3 | 7.3% | 3 | 8.8% | 3 | 9.7% | 1 | 5.9% | 0 | 0.0% |
| Tricuspid Inflow Signals: E and A | 77 | 16.2% | 29 | 11.7% | 17 | 17.3% | 6 | 14.6% | 7 | 20.6% | 13 | 41.9% | 5 | 29.4% | 0 | 0.0% |
| Tissue Doppler of the tricuspid annulus | 239 | 50.4% | 134 | 54.0% | 32 | 32.7% | 23 | 56.1% | 18 | 52.9% | 20 | 64.5% | 11 | 64.7% | 1 | 20.0% |
| Report RV E/e ratio | 37 | 7.8% | 14 | 5.6% | 10 | 10.2% | 4 | 9.8% | 4 | 11.8% | 2 | 6.5% | 3 | 17.6% | 0 | 0.0% |
| Comment on septal curvature or measurement of LVEI | 80 | 16.9% | 34 | 13.7% | 13 | 13.3% | 10 | 24.4% | 6 | 17.6% | 10 | 32.3% | 7 | 41.2% | 0 | 0.0% |
| Cardiac output in the RVOT | 66 | 13.9% | 21 | 8.5% | 22 | 22.4% | 9 | 22.0% | 6 | 17.6% | 5 | 16.1% | 3 | 17.6% | 0 | 0.0% |
| None of the above | 3 | 0.6% | 1 | 0.4% | 1 | 1.0% | 0 | 0.0% | 1 | 2.9% | 0 | 0.0% | 0 | 0.0% | 0 | 0.0% |

# Supplemental Table 3: Evaluation of Tricuspid Valve and Blood Conducting System by TTE

|  | **All responses N=474** | | **General non-invasive cardiology N=248** | | **Peri-operative care and anesthesia  N=98** | | **Intensive care setting  N=34** | | **Invasive echocardiography (procedures) N=34** | | **Congenital heart disease  N=31** | | **Pulmonary artery hypertension specialist  N=17** | | **Inherited cardiac conditions / Sports cardiology N=5** | |
| --- | --- | --- | --- | --- | --- | --- | --- | --- | --- | --- | --- | --- | --- | --- | --- | --- |
|  | Count | Percent | Count | Percent | Count | Percent | Count | Percent | Count | Percent | Count | Percent | Count | Percent | Count | Percent |
| **Tricuspid Valve (TTE)** | | | | | | | | | | | | | | | | |
| Annular dimensions | 191 | 40.3% | 77 | 31.0% | 56 | 57.1% | 16 | 39.0% | 18 | 52.9% | 17 | 54.8% | 6 | 35.3% | 1 | 20.0% |
| Grading of regurgitation severity | 399 | 84.2% | 222 | 89.5% | 68 | 69.4% | 31 | 75.6% | 29 | 85.3% | 30 | 96.8% | 15 | 88.2% | 4 | 80.0% |
| Measurement of TR Vena contracta | 251 | 53% | 131 | 52.8% | 51 | 52.0% | 22 | 53.7% | 26 | 76.5% | 13 | 41.9% | 7 | 41.2% | 1 | 20.0% |
| Right ventricular systolic pressure (RVSP) | 368 | 77.6% | 209 | 84.3% | 59 | 60.2% | 31 | 75.6% | 23 | 67.6% | 29 | 93.5% | 14 | 82.4% | 3 | 60.0% |
| None of the above | 6 | 1.3% | 0 | 0.0% | 5 | 5.1% | 1 | 2.4% | 0 | 0.0% | 0 | 0.0% | 0 | 0.0% | 0 | 0.0% |
| **Right atrium (TTE)** | | | | | | | | | | | | | | | | |
| Right atrial linear dimensions | 114 | 24.1% | 52 | 21.0% | 34 | 34.7% | 12 | 29.3% | 6 | 17.6% | 6 | 19.4% | 4 | 23.5% | 0 | 0.0% |
| Right atrial area | 243 | 51.3% | 143 | 57.7% | 23 | 23.5% | 22 | 53.7% | 21 | 61.8% | 19 | 61.3% | 12 | 70.6% | 3 | 60.0% |
| Right atrial volume | 133 | 28.1% | 75 | 30.2% | 16 | 16.3% | 9 | 22.0% | 17 | 50.0% | 9 | 29.0% | 6 | 35.3% | 1 | 20.0% |
| Right atrial strain | 24 | 5.1% | 10 | 4.0% | 3 | 3.1% | 1 | 2.4% | 4 | 11.8% | 1 | 3.2% | 4 | 23.5% | 1 | 20.0% |
| None of the above | 82 | 17.3% | 29 | 11.7% | 32 | 32.7% | 10 | 24.4% | 2 | 5.9% | 8 | 25.8% | 1 | 5.9% | 0 | 0.0% |
| **Pulmonary Valve and Pulmonary Artery (TTE)** | | | | | | | | | | | | | | | | |
| Comment on / measure pulmonary flow profiles * | 275 | 58% | 159 | 64.1% | 32 | 32.7% | 23 | 56.1% | 20 | 58.8% | 25 | 80.6% | 13 | 76.5% | 3 | 60.0% |
| Measure peak PR (estimate of mean pulmonary pressure) | 225 | 47.5% | 123 | 49.6% | 31 | 31.6% | 17 | 41.5% | 17 | 50.0% | 25 | 80.6% | 10 | 58.8% | 2 | 40.0% |
| Measure early-diastolic PR velocity | 90 | 19% | 43 | 17.3% | 13 | 13.3% | 7 | 17.1% | 8 | 23.5% | 12 | 38.7% | 7 | 41.2% | 0 | 0.0% |
| Measure end-diastolic pulmonary PR (estimate of diastolic pulmonary pressure) | 140 | 29.% | 78 | 31.5% | 18 | 18.4% | 11 | 26.8% | 8 | 23.5% | 17 | 54.8% | 8 | 47.1% | 0 | 0.0% |
| None of the above | 82 | 17.3% | 34 | 13.7% | 35 | 35.7% | 7 | 17.1% | 4 | 11.8% | 0 | 0.0% | 2 | 11.8% | 0 | 0.0% |
| **Venous excess ultrasound (VExUS) / Hepatic blood flow (TTE)** | | | | | | | | | | | | | | | | |
| Measure size of inferior caval vein (IVC) and collapse index | 353 | 74.5% | 204 | 82.3% | 57 | 58.2% | 32 | 78.0% | 24 | 70.6% | 24 | 77.4% | 11 | 64.7% | 1 | 20.0% |
| VExUS scoring | 41 | 8.6% | 9 | 3.6% | 15 | 15.3% | 10 | 24.4% | 2 | 5.9% | 2 | 6.5% | 3 | 17.6% | 0 | 0.0% |
| Hepatic vein flow profile | 217 | 45.8% | 113 | 45.6% | 49 | 50.0% | 23 | 56.1% | 15 | 44.1% | 12 | 38.7% | 5 | 29.4% | 0 | 0.0% |
| Portal vein flow profile | 47 | 9.9% | 18 | 7.3% | 18 | 18.4% | 8 | 19.5% | 1 | 2.9% | 1 | 3.2% | 1 | 5.9% | 0 | 0.0% |
| Interlobular vein flow profile (kidney) | 22 | 4.6% | 5 | 2.0% | 10 | 10.2% | 6 | 14.6% | 0 | 0.0% | 1 | 3.2% | 0 | 0.0% | 0 | 0.0% |
| None of the above | 55 | 11.6% | 21 | 8.5% | 16 | 16.3% | 3 | 7.3% | 4 | 11.8% | 6 | 19.4% | 2 | 11.8% | 3 | 60.0% |

* e.g. acceleration time, presence of a notch; PR: pulmonary regurgitation

# Supplemental Table 4: Dimensions and function by TEE

|  | Total N=424 | | Peri-operative care and anesthesia N=196 | | General non-invasive cardiology N=128 | | Invasive echocardiography (procedures) N=34 | | Intensive care setting N=29 | | Congenital heart disease N=22 | | Pulmonary artery hypertension specialist N=12 | | Inherited cardiac conditions / Sports cardiology N=3 | |
| --- | --- | --- | --- | --- | --- | --- | --- | --- | --- | --- | --- | --- | --- | --- | --- | --- |
|  | Count | Percent | Count | Percent | Count | Percent | Count | Percent | Count | Percent | Count | Percent | Count | Percent | Count | Percent |
| **Dimensions, Areas and Volumes** | | | | | | | | | | | | | | | | |
| RVD1 only | 100 | 23.6% | 39 | 19.9% | 32 | 25.0% | 10 | 29.4% | 9 | 31.0% | 6 | 27.3% | 4 | 33.3% | 0 | 0.0% |
| All RV linear dimensions inflow (RVD1, RVD2, RVD3) | 107 | 25.2% | 70 | 35.7% | 13 | 10.2% | 6 | 17.6% | 12 | 41.4% | 5 | 22.7% | 1 | 8.3% | 0 | 0.0% |
| RV linear dimensions outflow (RVOT) | 85 | 20.0% | 42 | 21.4% | 19 | 14.8% | 6 | 17.6% | 10 | 34.5% | 5 | 22.7% | 2 | 16.7% | 1 | 33.3% |
| RV end-diastolic and end-sysolic area | 109 | 25.7% | 72 | 36.7% | 16 | 12.5% | 6 | 17.6% | 12 | 41.4% | 2 | 9.1% | 1 | 8.3% | 0 | 0.0% |
| RV volumes by 3D echocardiography | 47 | 11.1% | 26 | 13.3% | 7 | 5.5% | 3 | 8.8% | 6 | 20.7% | 2 | 9.1% | 2 | 16.7% | 1 | 33.3% |
| RV wall thickness | 108 | 25.5% | 69 | 35.2% | 16 | 12.5% | 8 | 23.5% | 11 | 37.9% | 2 | 9.1% | 1 | 8.3% | 1 | 33.3% |
| None of the above | 142 | 33.5% | 39 | 19.9% | 69 | 53.9% | 11 | 32.4% | 4 | 13.8% | 12 | 54.5% | 6 | 50.0% | 1 | 33.3% |
| **Systolic and diastolic function** | | | | | | | | | | | | | | | | |
| Visual estimation of RV function | 295 | 69.6% | 153 | 78.1% | 78 | 60.9% | 18 | 52.9% | 22 | 75.9% | 19 | 86.4% | 4 | 33.3% | 1 | 33.3% |
| Tei Index | 13 | 3.1% | 8 | 4.1% | 2 | 1.6% | 1 | 2.9% | 1 | 3.4% | 1 | 4.5% | 0 | 0.0% | 0 | 0.0% |
| RV Fractional Area Change (FAC) | 144 | 34.0% | 99 | 50.5% | 18 | 14.1% | 10 | 29.4% | 10 | 34.5% | 5 | 22.7% | 2 | 16.7% | 0 | 0.0% |
| TAPSE by M-Mode | 185 | 43.6% | 124 | 63.3% | 26 | 20.3% | 10 | 29.4% | 13 | 44.8% | 7 | 31.8% | 3 | 25.0% | 2 | 66.7% |
| Report on TAPSE/RVSP | 75 | 17.7% | 53 | 27.0% | 11 | 8.6% | 3 | 8.8% | 4 | 13.8% | 2 | 9.1% | 1 | 8.3% | 1 | 33.3% |
| 2D-derived RV 4Ch-strain | 29 | 6.8% | 18 | 9.2% | 7 | 5.5% | 1 | 2.9% | 3 | 10.3% | 0 | 0.0% | 0 | 0.0% | 0 | 0.0% |
| 2D-derived RV free-wall-longitudinal strain | 46 | 10.8% | 24 | 12.2% | 9 | 7.0% | 5 | 14.7% | 6 | 20.7% | 1 | 4.5% | 1 | 8.3% | 0 | 0.0% |
| RV 3D ejection fraction | 42 | 9.9% | 24 | 12.2% | 4 | 3.1% | 4 | 11.8% | 6 | 20.7% | 1 | 4.5% | 3 | 25.0% | 0 | 0.0% |
| RV 3D motion decomposition / RV 3D strains | 12 | 2.8% | 3 | 1.5% | 4 | 3.1% | 0 | 0.0% | 3 | 10.3% | 1 | 4.5% | 1 | 8.3% | 0 | 0.0% |
| Tricuspid Inflow Signals: E and A | 51 | 12.0% | 34 | 17.3% | 8 | 6.3% | 2 | 5.9% | 3 | 10.3% | 4 | 18.2% | 0 | 0.0% | 0 | 0.0% |
| Tissue Doppler of the tricuspid annulus | 76 | 17.9% | 49 | 25.0% | 16 | 12.5% | 3 | 8.8% | 7 | 24.1% | 1 | 4.5% | 0 | 0.0% | 0 | 0.0% |
| Report RV E/e ratio | 22 | 5.2% | 15 | 7.7% | 6 | 4.7% | 1 | 2.9% | 0 | 0.0% | 0 | 0.0% | 0 | 0.0% | 0 | 0.0% |
| Comment on septal curvature or measurement of LVEI | 55 | 13.0% | 31 | 15.8% | 10 | 7.8% | 6 | 17.6% | 6 | 20.7% | 2 | 9.1% | 0 | 0.0% | 0 | 0.0% |
| Cardiac output in the RVOT | 60 | 14.2% | 36 | 18.4% | 11 | 8.6% | 4 | 11.8% | 7 | 24.1% | 2 | 9.1% | 0 | 0.0% | 0 | 0.0% |
| None of the above | 44 | 10.4% | 1 | 0.5% | 31 | 24.2% | 5 | 14.7% | 2 | 6.9% | 1 | 4.5% | 4 | 33.3% | 0 | 0.0% |

# Supplemental Table 5: Evaluation of Tricuspid Valve and Blood Conducting System by TEE

|  | **All responses N=424** | | **Peri-operative care and anesthesia N=196** | | **General non-invasive cardiology N=128** | | **Invasive echocardiography (procedures) N=34** | | **Intensive care setting  N=29** | | **Congenital heart disease N=22** | | **Pulmonary artery hypertension specialist N=12** | | **Inherited cardiac conditions / Sports cardiology N=3** | |
| --- | --- | --- | --- | --- | --- | --- | --- | --- | --- | --- | --- | --- | --- | --- | --- | --- |
|  | Count | Percent | Count | Percent | Count | Percent | Count | Percent | Count | Percent | Count | Percent | Count | Percent | Count | Percent |
| **Tricuspid Valve (TOE/TEE)** | | | | | | | | | | | | | | | | |
| Annular dimensions | 253 | 59.7% | 148 | 75.5% | 54 | 42.2% | 20 | 58.8% | 12 | 41.4% | 14 | 63.6% | 3 | 25.0% | 2 | 66.7% |
| Grading of regurgitation severity | 348 | 82.1% | 160 | 81.6% | 107 | 83.6% | 28 | 82.4% | 23 | 79.3% | 18 | 81.8% | 10 | 83.3% | 2 | 66.7% |
| Measurement of TR Vena contracta | 246 | 58.0% | 125 | 63.8% | 64 | 50.0% | 26 | 76.5% | 18 | 62.1% | 8 | 36.4% | 5 | 41.7% | 0 | 0.0% |
| Right ventricular systolic pressure (RVSP) | 265 | 62.5% | 130 | 66.3% | 78 | 60.9% | 18 | 52.9% | 18 | 62.1% | 17 | 77.3% | 3 | 25.0% | 1 | 33.3% |
| None of the above | 11 | 2.6% | 3 | 1.5% | 7 | 5.5% | 0 | 0.0% | 0 | 0.0% | 0 | 0.0% | 1 | 8.3% | 0 | 0.0% |
| **Right atrium (TOE/TEE)** | | | | | | | | | | | | | | | | |
| Right atrial linear dimensions | 108 | 25.5% | 69 | 35.2% | 23 | 18.0% | 3 | 8.8% | 6 | 20.7% | 4 | 18.2% | 2 | 16.7% | 1 | 33.3% |
| Right atrial area | 86 | 20.3% | 29 | 14.8% | 26 | 20.3% | 10 | 29.4% | 11 | 37.9% | 6 | 27.3% | 3 | 25.0% | 1 | 33.3% |
| Right atrial strain | 19 | 4.5% | 8 | 4.1% | 7 | 5.5% | 2 | 5.9% | 0 | 0.0% | 1 | 4.5% | 1 | 8.3% | 0 | 0.0% |
| None of the above | 225 | 53.1% | 92 | 46.9% | 82 | 64.1% | 17 | 50.0% | 12 | 41.4% | 15 | 68.2% | 7 | 58.3% | 0 | 0.0% |
| **Pulmonary Valve and Pulmonary Artery (TOE/TEE)** | | | | | | | | | | | | | | | | |
| Comment on / measure pulmonary flow profiles* | 137 | 32.3% | 65 | 33.2% | 29 | 22.7% | 10 | 29.4% | 16 | 55.2% | 14 | 63.6% | 1 | 8.3% | 2 | 66.7% |
| Measure peak PR velocity (estimate of mean pulmonary pressure) | 114 | 26.9% | 57 | 29.1% | 23 | 18.0% | 10 | 29.4% | 10 | 34.5% | 11 | 50.0% | 3 | 25.0% | 0 | 0.0% |
| Measure early-diastolic PR velocity | 43 | 10.1% | 20 | 10.2% | 9 | 7.0% | 4 | 11.8% | 5 | 17.2% | 3 | 13.6% | 1 | 8.3% | 1 | 33.3% |
| Measure end-diastolic PR velocity (estimate of diastolic pulmonary pressure) | 55 | 13.0% | 25 | 12.8% | 12 | 9.4% | 5 | 14.7% | 6 | 20.7% | 5 | 22.7% | 2 | 16.7% | 0 | 0.0% |
| None of the above | 196 | 46.2% | 82 | 41.8% | 78 | 60.9% | 14 | 41.2% | 8 | 27.6% | 7 | 31.8% | 7 | 58.3% | 0 | 0.0% |
| **Venous excess ultrasound (VExUS) / Hepatic blood flow** | | | | | | | | | | | | | | | | |
| Measure size of inferior caval vein (IVC) and collaps index | 131 | 30.9% | 66 | 33.7% | 32 | 25.0% | 11 | 32.4% | 12 | 41.4% | 6 | 27.3% | 2 | 16.7% | 2 | 66.7% |
| VExUS scoring | 15 | 3.5% | 8 | 4.1% | 2 | 1.6% | 0 | 0.0% | 4 | 13.8% | 1 | 4.5% | 0 | 0.0% | 0 | 0.0% |
| Hepatic vein flow profile | 147 | 34.7% | 105 | 53.6% | 15 | 11.7% | 11 | 32.4% | 10 | 34.5% | 4 | 18.2% | 2 | 16.7% | 0 | 0.0% |
| Portal vein flow profile | 36 | 8.5% | 25 | 12.8% | 2 | 1.6% | 2 | 5.9% | 4 | 13.8% | 1 | 4.5% | 2 | 16.7% | 0 | 0.0% |
| Interlobular vein flow profile | 11 | 2.6% | 7 | 3.6% | 1 | 0.8% | 1 | 2.9% | 1 | 3.4% | 1 | 4.5% | 0 | 0.0% | 0 | 0.0% |
| None of the above | 191 | 45.0% | 56 | 28.6% | 88 | 68.8% | 13 | 38.2% | 11 | 37.9% | 15 | 68.2% | 8 | 66.7% | 0 | 0.0% |

* (e.g. acceleration time, presence of a notch); PR: pulmonary regurgitation

# Supplemental Table 5: Availability of a protocol to optimize 3D acquisitions

| **Single answer option** | **Count** | **Percent** |
| --- | --- | --- |
| 3D TTE protocol | 48 | 9.4% |
| 3D TOE protocol | 32 | 6.3% |
| 3D TTE and 3D TOE protocol | 23 | 4.5% |
| No | 408 | 79.8% |
| Total | 511 | 100% |

# Supplemental Table 6: Definition of end-systolic frame in the presence of abnormal septal motion

| **Single answer option** | **Count** | **Percent** |
| --- | --- | --- |
| Smallest right ventricle | 158 | 34.9% |
| Smallest left ventricle | 46 | 10.2% |
| Post septal shift | 25 | 5.5% |
| Post septal shift when the tricuspid valve is not fully opened | 59 | 13.0% |
| None of the above | 44 | 9.7% |
| I don't know | 121 | 26.7% |
| Total | 453 | 100% |

# Supplemental Table 7: Report on image quality

|  | **No** | | **Yes** | | **Total** |
| --- | --- | --- | --- | --- | --- |
|  | Count | Percent | Count | Percent | Count |
| Right Ventricle | 135 | 32.1% | 285 | 67.9% | 420 |
| Doppler Signals | 171 | 40.7% | 249 | 59.3% | 420 |

# Supplemental Table 8: Reporting on RV shape /geometry

| **Single Answer Option** | **Count** | **Percent** |
| --- | --- | --- |
| Specific measurements to differentiate between spherical,  conical and regional changes. | 27 | 6.4% |
| Visual assessment. | 305 | 72.4% |
| Do not report. | 89 | 21.1% |
| Total | 421 | 100% |

# Supplemental Table 9: Reporting on regional wall motion abnormalities of the RV (e.g. McConnel's sign, regional RV strain)

| **Yes / No question** | **Count** | **Percent** |
| --- | --- | --- |
| No | 122 | 29% |
| Yes | 299 | 71% |
| Total | 421 | 100% |

# Supplemental Table 10: 54: About automatic segmentation methods: Do you routinely use automatic segmentation methods (non strain imaging) for the right heart in the laboratory, such as autoRV (Tomtec), LVivoRV, US2.ai or others?

| **Single answer option** | **Count** | **Percent** |
| --- | --- | --- |
| Yes | 61 | 11,0 |
| No | 490 | 88,3 |
| Other methods *other methods: EchoPAC, Ventripoint | 4 | 0,7 |
| Total | 555 | 100,0 |
| Missing | 56 |  |
| Total | 611 |  |

# Supplemental Table 11: Grading systems of RV function

|  | **No** | | **Yes** | | **Not applicable** | | **Total** |
| --- | --- | --- | --- | --- | --- | --- | --- |
|  | Count | Percent | Count | Percent | Count | Percent | Count |
| **Cardiac surgery** | 179 | 43.6% | 176 | 42.8% | 56 | 13.6% | 411 |
| **Myocardial disease versus**   **pressure or volume overloaded**   **conditions** | 222 | 54.4% | 135 | 33.1% | 51 | 12.5% | 408 |
| **Athletics** | 203 | 50.0% | 80 | 19.7% | 123 | 30.3% | 406 |
| **Pregnancy** | 206 | 51.0% | 68 | 16.8% | 130 | 32.2% | 404 |
| **Other patient groups*** | 215 | 55.0% | 28 | 7.2% | 148 | 37.9% | 391 |
| **No - I always use the same grading system** | 118 | 30.4% | 169 | 43.6% | 101 | 26.0% | 388 |

*other patient groups: Adult Congenital Heart Disease, Atrial Septal Defect, Cardiomyopathies, CHD, liver transplantation, obesity, pacemaker, pulmonary embolism, pulmonary hypertension, ≥ severe TR, systemic RV (ccTGA, TGA post atrial switch), TOF, Heart Transplantation, VAD.
