# Supplementary material for: International practices and variability in right heart echocardiography: results from the RVNet(Work) international survey
Source: Echo Res Pract. 2026 Jun 8;13:22. doi: 10.1186/s44156-026-00121-7 (PMC13245076; doi:10.1186/s44156-026-00121-7)
Supplement: Supplementary file 2 — Supplementary Material 2 [file 44156_2026_121_MOESM2_ESM.pdf]

RVNet(Work) survey : Clinical practice in right heart echocardiography and imaging

Basic information

0% Complete.

Which gender do you feel you belong to?

- ◯ female
- ◯ male
- ◯ non-binary
- ◯ do not want to tell

In which country do you work?

- ◯ Afghanistan
- ◯ Albania
- ◯ Algeria
- ◯ Andorra
- ◯ Angola
- ◯ Antigua and Barbuda
- ◯ Argentina
- ◯ Armenia
- ◯ Australia
- ◯ Austria
- ◯ Azerbaijan
- ◯ The Bahamas
- ◯ Bahrain
- ◯ Bangladesh
- ◯ Barbados
- ◯ Belarus
- ◯ Belgium
- ◯ Belize
- ◯ Benin
- ◯ Bhutan
- ◯ Bolivia
- ◯ Bosnia and Herzegovina
- ◯ Botswana
- ◯ Brazil
- ◯ Brunei
- ◯ Bulgaria
- ◯ Burkina Faso
- ◯ Burundi
- ◯ Cabo Verde
- ◯ Cambodia
- ◯ Cameroon
- ◯ Canada
- ◯ Central African Republic
- ◯ Chad
- ◯ Chile
- ◯ China
- ◯ Colombia
- ◯ Comoros
- ◯ Congo (Democratic Republic of the)
- ◯ Congo (Republic of the)
- ◯ Costa Rica
- ◯ Côte d'Ivoire
- ◯ Croatia
- ◯ Cuba
- ◯ Cyprus
- ◯ Czech Republic
- ◯ Denmark
- ◯ Djibouti
- ◯ Dominica
- ◯ Dominican Republic
- ◯ East Timor (Timor-Leste)
- ◯ Ecuador
- ◯ Egypt
- ◯ El Salvador
- ◯ Equatorial Guinea
- ◯ Eritrea
- ◯ Estonia
- ◯ Eswatini
- ◯ Ethiopia
- ◯ Fiji
- ◯ Finland
- ◯ France
- ◯ Gabon
- ◯ The Gambia
- ◯ Georgia
- ◯ Germany
- ◯ Ghana
- ◯ Greece
- ◯ Grenada

◯ Guatemala
◯ Guinea
◯ Guinea-Bissau
◯ Guyana
◯ Haiti
◯ Honduras
◯ Hungary
◯ Iceland
◯ India
◯ Indonesia
◯ Iran
◯ Iraq
◯ Ireland
◯ Israel
◯ Italy
◯ Jamaica
◯ Japan
◯ Jordan
◯ Kazakhstan
◯ Kenya
◯ Kiribati
◯ Korea (North)
◯ Korea (South)
◯ Kosovo
◯ Kuwait
◯ Kyrgyzstan
◯ Laos
◯ Latvia
◯ Lebanon
◯ Lesotho
◯ Liberia
◯ Libya
◯ Liechtenstein
◯ Lithuania
◯ Luxembourg
◯ Madagascar
◯ Malawi
◯ Malaysia
◯ Maldives
◯ Mali
◯ Malta
◯ Marshall Islands
◯ Mauritania
◯ Mauritius
◯ Mexico
◯ Micronesia (Federated States of)
◯ Moldova
◯ Monaco
◯ Mongolia
◯ Montenegro
◯ Morocco
◯ Mozambique
◯ Myanmar (Burma)
◯ Namibia
◯ Nauru
◯ Nepal
◯ Netherlands
◯ New Zealand
◯ Nicaragua
◯ Niger
◯ Nigeria
◯ North Macedonia
◯ Norway
◯ Oman
◯ Pakistan
◯ Palau
◯ Panama
◯ Papua New Guinea
◯ Paraguay
◯ Peru
◯ Philippines

◯ Poland
◯ Portugal
◯ Qatar
◯ Romania
◯ Russia
◯ Rwanda
◯ Saint Kitts and Nevis
◯ Saint Lucia
◯ Saint Vincent and the Grenadines
◯ Samoa
◯ San Marino
◯ Sao Tome and Principe
◯ Saudi Arabia
◯ Senegal
◯ Serbia
◯ Seychelles
◯ Sierra Leone
◯ Singapore
◯ Slovakia
◯ Slovenia
◯ Solomon Islands
◯ Somalia
◯ South Africa
◯ Spain
◯ Sri Lanka
◯ Sudan
◯ South
◯ Suriname
◯ Sweden
◯ Switzerland
◯ Syria
◯ Taiwan
◯ Tajikistan
◯ Tanzania
◯ Thailand
◯ Togo
◯ Tonga
◯ Trinidad and Tobago
◯ Tunisia
◯ Turkey
◯ Turkmenistan
◯ Tuvalu
◯ Uganda
◯ Ukraine
◯ United Arab Emirates
◯ United Kingdom
◯ United States
◯ Uruguay
◯ Uzbekistan
◯ Vanuatu
◯ Vatican City
◯ Venezuela
◯ Vietnam
◯ Yemen
◯ Zambia
◯ Zimbabwe

Practice related questions

4% Complete.

What is your profession?	◯ Physician<br>◯ Researcher<br>◯ Sonographer
What is the setting of your practice or activity?	◯ Academic hospital setting (e.g. university hospital)<br>◯ Non-academic hospital setting/clinic<br>◯ Outpatient<br>◯ Clinical research laboratory (mainly)

Footnote:

- United Kingdom
Academic hospital setting = Tertiary center
Non-academic hospital setting = District General Hospital
Outpatient = Community / Primary Care / Screening

- Germany / Deutschland
Academic hospital setting = Universitätsklinikum
Non-academic hospital setting = Krankenhaus (keine Universitätsklinik)
Outpatient = Niederlassung

What best describes your practice area?	◯ Peri-operative care and anesthesia<br>◯ Invasive echocardiography (procedures)<br>◯ General non-invasive cardiology<br>◯ Congenital heart disease<br>◯ Intensive care setting<br>◯ Pulmonary artery hypertension specialist<br>◯ Inherited cardiac conditions / Sports cardiology
Is the laboratory/institution where you work accredited by a quality society (e.g. IAC, EACVI, other national society)?	◯ Yes<br>◯ No<br>◯ not applicable (n/a)<br>◯ I don't know
Are you "echo" accredited or certified?	◯ Echo board certification<br>◯ Accreditation by Echo society<br>◯ No
How many years have you practiced echocardiography?	◯ less than 5 years<br>◯ 5 - 10 years<br>◯ 10 - 20 years<br>◯ greater than 20 years
Do you perform transthoracic echocardiography (TTE)?	◯ Yes<br>◯ No
Please estimate the number of TTE examinations you carry out per week.	______________________

Please check whether the entered value is correct.

Do you perform transoesophageal echocardiography (TEE/TOE)?	◯ Yes<br>◯ No
Please estimate the number of TOE/TEE examinations you carry out per week.	__________________________

Please check whether the entered value is correct.

Equipment and methods used in the laboratory/setting

17% Complete.

Which machines do you routinely use?

- ☐ Philips
- ☐ General Electric (GE)
- ☐ Siemens
- ☐ other vendors: please specify below

Other vendors:

____

Who mainly acquires the images in your setting/laboratory?

- ☐ sonographers
- ☐ cardiology fellows
- ☐ practicing cardiologist / clinician / researcher
- ☐ cardiovascular anaesthesiologist

Which parameters do you routinely acquire or measure by transthoracic echocardiography (TTE)?

Right ventricle (TTE) : Dimensions, Areas and Volumes

- ☐ RVD1 only
- ☐ all RV linear dimensions "inflow" (RVD1, RVD2, RVD3)
- ☐ RV linear dimensions "outflow" (RVOT)
- ☐ RV end-diastolic and end-sysolic area
- ☐ RV volumes by 3D echocardiography
- ☐ RV wall thickness
- ☐ None of the above

You have selected an answer and also 'none of the above'. Are you sure?

Right Ventricle (TTE) : Systolic and diastolic function

- ☐ Visual estimation of RV function
- ☐ Tei Index
- ☐ RV Fractional Area Change (FAC)
- ☐ TAPSE by M-Mode
- ☐ TAPSE/RVSP
- ☐ 2D-derived RV 4Ch-strain
- ☐ 2D-derived RV free-wall-longitudinal strain
- ☐ RV 3D ejection fraction
- ☐ RV 3D motion decomposition / RV 3D strains
- ☐ Tricuspid Inflow Signals: E and A
- ☐ Tissue Doppler of the tricuspid annulus
- ☐ Report RV E/e' ratio
- ☐ comment on septal curvature or measurement of LV eccentricity index
- ☐ cardiac output in the RVOT
- ☐ None of the above

You have selected an answer and also 'none of the above'. Are you sure?

Tricuspid Valve (TTE)

- ☐ Annular dimensions
- ☐ Grading of regurgitation severity
- ☐ Measurement of TR Vena contracta
- ☐ Right ventricular systolic pressure (RVSP)
- ☐ None of the above

You have selected an answer and also 'none of the above'. Are you sure?

Right atrium (TTE)	☐ Right atrial linear dimensions<br>☐ RIght atrial area<br>☐ Right atrial volume<br>☐ Right atrial strain<br>☐ None of the above
You have selected an answer and also 'none of the above'. Are you sure?	
Pulmonary Valve and Pulmonary Artery (TTE)	☐ Comment on / measure pulmonary flow profiles (e.g. acceleration time, presence of a notch)<br>☐ Measure peak pulmonary regurgitation velocity (estimate of mean pulmonary pressure)<br>☐ Measure early-diastolic pulmonary regurgitation velocity<br>☐ Measure end-diastolic pulmonary regurgitation velocity (estimate of diastolic pulmonary pressure)<br>☐ None of the above
You have selected an answer and also 'none of the above'. Are you sure?	
Venous excess ultrasound (VExUS) / Hepatic blood flow (TTE)	☐ Measure size of inferior caval vein (IVC) and collapse index<br>☐ VExUS scoring<br>☐ Hepatic vein flow profile<br>☐ Portal vein flow profile<br>☐ Interlobular vein flow profile (kidney)<br>☐ None of the above
You have selected an answer and also 'none of the above'. Are you sure?	
Which parameters do you routinely acquire or measure by transoesophageal echocardiography (TOE/TEE)?	
Right ventricle (TOE/TEE) : Dimensions, Areas and Volumes	☐ RVD1 only<br>☐ all RV linear dimensions "inflow" (RVD1, RVD2, RVD3)<br>☐ RV linear dimensions "outflow" (RVOT)<br>☐ RV end-diastolic and end-sysolic area<br>☐ RV volumes by 3D echocardiography<br>☐ RV wall thickness<br>☐ None of the above
You have selected an answer and also 'none of the above'. Are you sure?	

Right Ventricle (TOE/TEE) : Systolic and diastolic function

- ☐ Visual estimation of RV function
- ☐ Tei Index
- ☐ RV Fractional Area Change (FAC)
- ☐ TAPSE by M-Mode
- ☐ Report on TAPSE/RVSP
- ☐ 2D-derived RV 4Ch-strain
- ☐ 2D-derived RV free-wall-longitudinal strain
- ☐ RV 3D ejection fraction
- ☐ RV 3D motion decomposition / RV 3D strains
- ☐ Tricuspid Inflow Signals: E and A
- ☐ Tissue Doppler of the tricuspid annulus
- ☐ Report RV E/e' ratio
- ☐ Comment on septal curvature or measurement of LV eccentricity index
- ☐ Cardiac output in the RVOT
- ☐ None of the above

You have selected an answer and also 'none of the above'. Are you sure?

Tricuspid Valve (TOE/TEE)

- ☐ Annular dimensions
- ☐ Grading of regurgitation severity
- ☐ Measurement of TR Vena contracta
- ☐ Right ventricular systolic pressure (RVSP)
- ☐ None of the above

You have selected an answer and also 'none of the above'. Are you sure?

Right atrium (TOE/TEE)

- ☐ Right atrial linear dimensions
- ☐ RIght atrial area
- ☐ Right atrial strain
- ☐ None of the above

You have selected an answer and also 'none of the above'. Are you sure?

Pulmonary Valve and Pulmonary Artery (TOE/TEE)

- ☐ Comment on / measure pulmonary flow profiles (e.g. acceleration time, presence of a notch)
- ☐ Measure peak pulmonary regurgitation velocity (estimate of mean pulmonary pressure)
- ☐ Measure early-diastolic pulmonary regurgitation velocity
- ☐ Measure end-diastolic pulmonary regurgitation velocity (estimate of diastolic pulmonary pressure)
- ☐ None of the above

You have selected an answer and also 'none of the above'. Are you sure?

Venous excess ultrasound (VExUS) / Hepatic blood flow (TOE/TEE)

- ☐ Measure size of inferior caval vein (IVC) and collaps index
- ☐ VExUS scoring
- ☐ Hepatic vein flow profile
- ☐ Portal vein flow profile
- ☐ Interlobular vein flow profile
- ☐ None of the above

You have selected an answer and also 'none of the above'. Are you sure?

About automatic segmentation methods:<br>Do you routinely use automatic segmentation methods (non strain imaging) for the right heart in the laboratory, such as autoRV (Tomtec), LVivoRV, US2.ai or others?	◯ Yes<br>◯ No<br>◯ Other method(s) - please specify below
Other automatic segmentation method used:	__________________________________

View acquisitions and measures of the right ventricle

26% Complete.

TTE: Which transthoracic views do you routinely acquire by transthoracic echocardiography?

- ☐ Apical 4-chamber view
- ☐ RV-focused apical 4-chamber view
- ☐ Modified apical 4-chamber view (RV modified view)
- ☐ Parasternal long axis RV inflow view
- ☐ Parasternal long axis RV outflow view
- ☐ Parasternal short axis view focus on pulmonary valve
- ☐ Parasternal short axis view focus on tricuspid valve
- ☐ None of the above

You have selected an answer and also 'none of the above'. Are you sure?

Note:
Wording according to the 2019 Guidelines for Performing a Comprehensive Transthoracic Echocardiographic Examination in Adults: Recommendations from the American Society of Echocardiograph (DOI: 10.1016/j.echo.2018.06.004).

TTE: In which view do you usually acquire the M-mode for measuring TAPSE?

- ◯ Standard apical 4 chamber view
- ◯ RV focused view
- ◯ RV modified view optimizing alignment with annular excursion
- ◯ RV focused view using anatomical M-mode

TTE: If you have a 3D probe available: Do you use electronic sound angle modifciation technology (e.g. iRotate) when identifying the RV focused view?

- ◯ Yes
- ◯ No
- ◯ No 3D probe available

TOE/TEE: Which views do you routinely acquire by transoesophageal echocardiography ?

- ☐ Mid-esophageal 4-chamber view
- ☐ RV focused mid-esophageal 4-chamber view
- ☐ Mid-esophageal RV inflow-outflow
- ☐ Mid-esophageal modified bicaval view
- ☐ Transgastric RV inflow
- ☐ None of the above

You have selected an answer and also 'none of the above'. Are you sure?

Note:
Wording according to the 2013 Guidelines for Performing a Comprehensive Transesophageal Echocardiographic Examination: Recommendations from the American Society of Echocardiography and the Society of Cardiovascular Anesthesiologistsh (DOI: 10.1016/j.echo.2013.07.009).

TEE/TOE : In which view do you usually acquire images/loops for measuring TAPSE?

- ◯ Standard ME 4 chamber view (anatomical M-Mode)
- ◯ ME RV focused view (anatomical M-Mode)
- ◯ Transgastric RV inflow-outflow-view
- ◯ Other view
- ◯ I do not measure TAPSE with TEE/TOE.

Do you have a protocol to optimize 3D acquisitions of the right ventricle?

- ◯ 3D TTE protocol
- ◯ 3D TOE protocol
- ◯ 3D TTE and 3D TOE protocol
- ◯ No

Image analyses questions

36% Complete.

How do you define end-diastole for measures of the right ventricle?

- ◯ At the tricuspid valve closure
- ◯ One frame before the tricuspid valve closure
- ◯ Largest right ventricle
- ◯ Highest lateral tricuspid annular plane
- ◯ At the beginning of the QRS
- ◯ At the peak of the QRS
- ◯ Other combination please specify

Other combination

____

How do you define end-systole for measures of the right ventricle?

- ◯ at the tricuspid valve opening
- ◯ one frame before the tricuspid valve opening
- ◯ smallest right ventricle
- ◯ lowest lateral tricuspid annular plane
- ◯ none of the above

In the presence of abnormal septal motion, how do you define the end-systolic frame?

- ◯ smallest right ventricle
- ◯ smallest left ventricle
- ◯ post septal shift
- ◯ post septal shift when the tricuspid valve is not fully opened
- ◯ none of the above
- ◯ I don't know

What would be your theoretical preference to measure the blood-myocardium interface (border between blood and myocardium) of the right ventricle?

- ◯ at the non-compacted region
- ◯ at the compacted region
- ◯ other - please specify below

green line: compacted region;
orange dotted line: non-compacted region

Other

____

Would you use the same criteria for the blood-myocardium interface of the right ventricle in systole and diastole?

◯ Yes ◯ No

Which reference centerline should be used to standardize transverse measures of the right ventricle?

- ◯ Linear centerline (from mid annulus to RV apex) as usually done
- ◯ A non-linear centerline that follows the curvature of the right ventricle (similar to what is done for the aorta or coronary arteries)
- ◯ Parallel measures to the tricuspid annulus independent of the centerline
- ◯ Other - please specify below

Other

____

How would you define the right ventricular apex?<br>(as this has implications for myocardial strain measures and segmental wall analysis)<br><br>Green dot: Apex positioned at the septal junction	◯ Apex positioned at the septal junction<br>◯ Apex defined as the maximal distance from the right ventricular centroid to the RV contour<br>◯ I don't know
When analyzing blood Doppler signals do you use the modal frequency (best defined signal) for reporting maximal velocity?<br><br>green line / cross: modal frequency	◯ Yes ◯ No
When analyzing tissue Doppler signals do you use the modal frequency for reporting maximal velocity?<br><br>green line / cross: modal frequency	◯ Yes ◯ No

Reporting and interpretation of acquired images

57% Complete.

How do you index (scale) right ventricular or right atrial size measures?

	No index	Index to body surface area (BSA)	Index to height	Index to a different coefficient
RV diameters	○	○	○	○
RV areas	○	○	○	○
RV volumes	○	○	○	○

What do you use to report on right ventricular shape / geometry?

- ○ Specific measurements to differentiate between spherical, conical and regional changes.
- ○ Visual assessment.
- ○ Do not report.

Do you report on regional wall motion abnormalities of the RV (e.g. McConnel's sign, regional RV strain)?

- ○ Yes
- ○ No

Do you use a standardized system / standardized cut-off values....

	Yes	No
...to grade the overall severity of RV dysfunction?	○	○
...to grade RV function by TAPSE	○	○
...to grade RV function by FAC	○	○
...to grade RV function by FWLS	○	○
...to grade RV function by 3D-EF	○	○

We invite you to comment on your grading system of RV function. Please use the text-box.

____

Do you use different parameters/combination of parameters for the grading of the RV according to the underlying condition/situation?

	Yes	No	Not applicable
Cardiac surgery	○	○	○
Myocardial disease versus pressure or volume overloaded conditions	○	○	○
Athletics	○	○	○
Pregnancy	○	○	○
Other patient groups - please specify below	○	○	○
No - I always use the same grading system	○	○	○

Other patient groups

____

In estimating right atrial pressure, in addition to inferior caval vein size/collapsibility or CVP, do you routinely report on

- ☐ Hepatic vein flow
- ☐ Portal vein flow
- ☐ Measure tricuspid E/e' ratio
- ☐ None of the above

You have selected an answer and also 'none of the above'. Are you sure?

Does your laboratory comment on right heart adaptation profiles using a scoring system?

- ◯ Yes
- ◯ No
- ◯ Not relevant in my field of practice

Does your reporting system include probability of syndrome or disease, example comment on probability of pulmonary hypertension or pulmonary vascular disease?

- ◯ Yes
- ◯ No
- ◯ Not relevant in my field of practice

Does your institution / laboratory have established criteria for reference change of values or what is judged as a meaningful change in right ventricular systolic pressure (RVSP) or RV systolic function?

◯ Yes ◯ No

Do you routinely report and comment on image quality of the right ventricle or tricuspid regurgitation signals?

	Yes	No
Right Ventricle	◯	◯
Doppler Signals	◯	◯

Do you use / does you institution use deep learning technology or machine learning technology to assist with:

	Yes	No
Prognostic scores	◯	◯
Diagnosis of syndromes (like pulmonary hypertension)	◯	◯

Specific pathology-based questions

75% Complete.

Does right heart imaging add value in risk stratification in in pulmonary arterial hypertension?	◯ Yes<br>◯ Minimally<br>◯ No
Which parameter/metric do you consider the most prognostic in pulmonary arterial hypertension?	◯ RV free wall strain<br>◯ TAPSE<br>◯ TAPSE/RVSP<br>◯ RVFAC<br>◯ RV myocardial performance index<br>◯ RV end-systolic dimension<br>◯ RVSP/PASP<br>◯ Pericardial effusion<br>◯ I don't know
Which metric do you consider the most prognostic in Tetralogy of Fallot?	◯ RV free wall strain<br>◯ TAPSE<br>◯ RVFAC<br>◯ RV myocardial performance index<br>◯ RV size<br>◯ RVSP/PASP<br>◯ Pericardial effusion<br>◯ I don't know
In endurance exercise training, it is very common to observe ...	◯ RV enlargement<br>◯ Right atrial enlargement<br>◯ Both right ventricular and atrial enlargement<br>◯ I don't know
In the early phase after cardiac surgery, it is common to observe ...	☐ Reduced overall right ventricular function<br>☐ Reduced longitudinal function of the RV<br>☐ Increased circumferential function of the RV<br>☐ No change in overall RV function<br>☐ I don't know

You have selected an answer and also 'I don't know'. Are you sure?

Multimodality imaging and diagnostic of the right heart

85% Complete.

Do you commonly use cardiac magnetic resonance of the right heart at your center?
- ◯ Yes
- ◯ No

Which methods do you routinely use for right ventricular assessment by MRI?
- ◯ Delayed enhancement
- ◯ T1 imaging
- ◯ 4D flow imaging

Do you commonly use computer tomography (CT) for right heart imaging at your center?
- ◯ Yes
- ◯ No

When measuring RV volumes do you include or exclude the non-compacted region for MRI or CT based studies?
- ◯ Include
- ◯ Exclude

green line: compacted region;
orange dotted line: non-compacted region

Do you routinely use right heart catheterization at your center?
- ◯ Yes
- ◯ No

Do you routinely use a pulmonary artery catheter in the intraoperative setting in patients with pulmonary hypertension or right ventricular dysfunction?
- ◯ Yes
- ◯ No

Please estimate the percentage (%) of patients undergoing cardiac surgery who receive a pulmonary catheter

____

Do you routinely use a pulmonary artery catheter in ICU in patients with pulmonary hypertension or right ventricular dysfunction?
- ◯ Yes
- ◯ No

Please estimate the percentage (%) of ICU patients who receive a pulmonary catheter

____

Collaborative questions

96% Complete.

Do you use a federated learning platform to collaborate with other centers focused on right heart imaging?	◯ Yes<br>◯ No
What is federated learning? Please see Wikipedia	
If not - would you be interested in joining a federated learning initiative where the data stays at the local center?	◯ Yes<br>◯ No
Please feel free to provide constructive comments to the RVNet(Work) group...	____
